# The Galectin Family in Colorectal Cancer: Integrating Molecular Mechanisms with Diagnostic, Prognostic, and Therapeutic Perspectives

**DOI:** 10.3390/biomedicines14091908

**Published:** 2026-08-26

**Authors:** Krystian Kozak, Monika Zajkowska

**Affiliations:** 1 ALAB Laboratoria sp. z o.o., Stępińska 22/30, 00-739 Warsaw, Poland; 2 Department of Neurodegeneration Diagnostics, Medical University of Białystok, 15-269 Białystok, Poland; monika.zajkowska@umb.edu.pl; 3 Department of Biochemical Diagnostics, Medical University of Bialystok Clinical Hospital, 15-269 Białystok, Poland

**Keywords:** CRC, Gal-1, Gal-2, Gal-3, Gal-4, Gal-7, Gal-8, Gal-9, Gal-10, Gal-12

## Abstract

**Background**: Colorectal cancer (CRC) remains one of the leading causes of cancer-related morbidity and mortality worldwide. Increasing evidence indicates that alterations in glycosylation and glycan-binding proteins, such as galectins, contribute substantially to colorectal carcinogenesis. We review the current knowledge on the role of known human galectins in CRC to provide up-to-date summaries, focusing on their involvement in tumor initiation, progression, invasion, angiogenesis, immune evasion, and treatment resistance, integrating molecular mechanisms with diagnostic, prognostic, and therapeutic perspectives. **Methods**: We searched for “colorectal cancer” and “Galectin”, “Galectin-X” and “colorectal cancer”, and “Gal-X” and “colorectal cancer” (X denotes the number of the respective galectin) in PubMed, ScienceDirect, Scopus, and Web of Science databases. All records were screened for relevance by the authors. **Results**: We summarize the expression patterns of individual galectins in colorectal tissues and circulation, their involvement in oncogenic signaling pathways, and their interactions with the tumor microenvironment. Furthermore, we critically evaluate the available evidence regarding their diagnostic, prognostic, and therapeutic potential. **Conclusions**: Although galectin-1 and galectin-3 are the most extensively investigated galectins, accumulating evidence suggests that galectin-4, galectin-8, and galectin-9 also play important context-dependent roles in CRC progression and immune regulation. However, methodological heterogeneity and limited clinical validation currently preclude the implementation of galectins as standalone biomarkers in routine practice. Future research should focus on standardized multicenter studies, clarification of context-dependent galectin functions, and the development of galectin-targeted therapies. A deeper understanding of galectin biology may facilitate the development of novel multimarker diagnostic approaches and personalized therapies for CRC patients.

## 1. Introduction

### 1.1. Colorectal Cancer

Colorectal cancer (CRC) is predominantly represented by adenocarcinoma, which usually originates from the glandular epithelial cells of the large intestine, as described in a population-based epidemiological review [1]. According to several narrative reviews, CRC is a heterogeneous group of diseases with various underlying genetic foundations and molecular pathways that influence individual susceptibility to cancer and have the ability to alter responsiveness to or induce resistance to antitumor treatments [2,3,4]. Contemporary reviews of colorectal carcinogenesis attributed CRC development to the accumulation of genetic and epigenetic mutations, which transform normal glandular epithelial cells into benign neoplasms (adenomas) [3,5]. Progression of tubulovillous and tubular adenomas into invasive carcinomas has long been recognized [3]. A review of CRC molecular biology also shows that hyperplastic polyps can progress to serrated adenomas with the potential for malignant transformation [5]. The 5-year survival rate of CRC is highly dependent on the stage. Statistics released in 2016 revealed that, in the USA, the 5-year survival rate was about 92% for stage I, and 12% for stage IV [1]. According to GLOBOCAN 2024 estimates, CRC constituted 9.9% of new cancer cases worldwide and reached a 9.4% mortality rate, corresponding to 2,041,017 new cases and 917,895 deaths. These data indicate that CRC is the third most commonly diagnosed cancer and the second leading cause of cancer death [6]. Surgery remains the primary treatment after early detection of localized disease [7]. In cases of metastasis (approximately 25% of CRC diagnoses [1]), surgery may no longer be effective. However, this percentage encompasses both resectable and unresectable metastases, and resection can still offer curative or disease-controlling benefit in amenable oligometastatic cases [8]. In patients with unresectable metastatic disease, the efficacy of cytotoxic therapies may be attenuated by the rapid induction of drug resistance and cancer recurrence, hence the need to research not only novel approaches to CRC treatment, but also its earlier detection [4].

#### Signaling Pathways in CRC

Molecular, cellular, and histological alterations in normal epithelium caused by both genetic and non-genetic events initiate the transition to adenoma and ultimately to cancer [9]. Genetic changes in CRC are mainly a result of genomic instability, which manifests in three main ways: chromosomal instability (CIN), microsatellite instability (MSI), and CpG island methylator phenotype (CIMP) [10,11]. These alterations can appear separately or in combination. CIMP commonly pairs with MSI due to the methylation and silencing of DNA mismatch repair genes [12]. Numerous genetic alterations can be found in CRC. However, many molecular changes are contained in a few signaling pathways. In a large-scale genomic and bioinformatic profiling analysis of CRC tumor samples (TCGA–The Cancer Genome Atlas Network), the Wnt pathway was the most frequently affected, with alterations in 93% [13] of tumors. This percentage reflects the cumulative burden of mutations across multiple Wnt components [13]. Inactivation of APC, one of the most commonly mutated tumor suppressor genes [14], initiates polyp-cancer progression [15]. Alterations in the PI3K/AKT/mTOR [16,17], JAK/STAT [18,19], and Hedgehog [20] signaling pathways have also been recognized as molecular mechanisms involved in CRC pathogenesis. Enhanced Wnt pathway activity inhibits the phosphorylation and subsequent degradation of β-catenin. A transcriptional complex composed of stabilized β-catenin and TCF/LEF with additional co-regulators such as Pygo and Bcl-9 induces the expression of Wnt target genes, including c-Myc and cyclin D1. This leads to increased CRC cell proliferation and disease progression. However, the effects of APC gene alterations are not limited to the Wnt pathway. APC inactivation may promote the Notch signaling pathway, which can further modulate Wnt signaling, and affect cell proliferation, stem cell maintenance, and apoptosis [21]. According to a review of TCGA genomic data, hypermutated tumors (an elevated rate of mutations) tend to have more mutations in TGF-β pathway genes [22]. Type II TGF-β receptor (TGFBR2) mutations can be found in 28% [23] of all CRC cases. Other mutations and epigenetic alterations found in CRC, affecting TGF-β signaling pathway members, include ACVR2A, SMAD4, ELF3 [22], and TSP1 [24]. On the other hand, non-hypermutated tumors show alterations mainly in p53 pathway genes [22]. KRAS is a proto-oncogene and a downstream effector of the epidermal growth factor receptor. It activates the MAPK pathway through BRAF signaling. Mutations in KRAS or BRAF induce proliferation and suppress apoptosis by aberrantly activating the MAPK signaling pathway. The prevalence of KRAS mutations in CRC is estimated at 30–50%. BRAF mutations, on the other hand, can be found in about 5–10% of CRC. Interestingly, KRAS and BRAF mutations are almost, but not entirely, mutually exclusive [25]. Taken together, these signaling pathways constitute a highly interrelated regulatory network that plays an important role in colorectal carcinogenesis and disease progression. Within this complex landscape, galectins have emerged as multifunctional modulators that intersect with key oncogenic axes (including Wnt/β-catenin, PI3K/AKT/mTOR, MAPK, and TGF-β), thereby influencing tumor cell behavior and tumor–microenvironment interactions.

### 1.2. Galectins

Galectins are a group of proteins that bind β-galactosides, primarily lactose and N-acetyllactosamine (LacNAc) [26,27]. Numerous review articles indicate that galectins, as a subgroup of lectins, selectively and reversibly bind carbohydrate moieties without exhibiting antibody, enzyme, or transporter characteristics, and without modifying the sugars themselves [27,28]. Extracellular galectins influence cellular behavior through multivalent binding to cell-specific glycans present on the cell surface [26,29]. Unlike other glycan-binding proteins, galectins have also been reported intracellularly, and have been described in a review article as the only protein family with known intracellular carbohydrate-binding activity [26]. Reviews also show that galectins can interact with intracellular proteins through glycan-independent mechanisms [26,30]. Mechanistic studies show that galectins are secreted from cells by an endoplasmic reticulum–Golgi-independent [31,32] pathway following cell activation, differentiation, or injury [26]. Physiologically, galectins may play a role in regulating immune responses, driving inflammatory processes, and modulating signaling [30] and autophagy [33]. Moreover, a recent review of human lectins literature has demonstrated that galectins are involved in tumor biology, contributing to carcinogenesis, cancer progression, metastasis, and angiogenesis [34].

This not only highlights their biological importance but also opens prospects for the use of selected galectins as biomarkers [35], as well as therapeutic targets for small-molecule inhibitors, monoclonal antibodies, and combination immunotherapies [36,37]. However, most of the evidence remains investigational and derives from preclinical models or early-phase trials. To date, no galectin-based biomarker or therapeutic strategy has been validated in a prospective clinical study.

This review aims to determine how individual members of the currently recognized human galectins influence colorectal cancer biology, how strong the available clinical evidence is for their use as biomarkers, and whether experimental data support their development as therapeutic targets, based on the current knowledge of galectin involvement in tumor initiation, progression, invasion, angiogenesis, immune evasion, and treatment resistance. It differs fundamentally from previously published reviews [38,39,40] through its family-wide comparison of galectins, its integration of intracellular and extracellular galectin functions, its emphasis on cellular compartments and the tumor microenvironment, and its investigation of multimarker strategies incorporating galectins in CRC.

## 2. Methods

To summarize the current evidence on galectins in CRC, a literature search was performed in PubMed, ScienceDirect, Scopus, and Web of Science databases. The search was conducted between 19 and 30 December 2025 and included studies published before January 2026, with the exception of two subsequently published articles [6,8] identified during the final verification of the manuscript and reference list before submission. The following search terms were used: “colorectal cancer” and “Galectin”, “Galectin-X” and “colorectal cancer”, and “Gal-X” and “colorectal cancer”, where X denotes the number of the respective galectin. Studies were included if they addressed CRC and galectins and were relevant to the biological, diagnostic, prognostic, or therapeutic aspects discussed in this review. CRC-specific evidence was prioritized during study selection. Where evidence from colorectal cancer was limited or unavailable, relevant findings from other cancer types or experimental models were considered to provide complementary biological context and identify potential mechanisms or implications for colorectal cancer. Such non-CRC evidence was used to complement, rather than replace, CRC-specific evidence and was interpreted with appropriate caution when drawing conclusions regarding CRC. Publications available in full text and relevant to the scope of the review were included, whereas duplicates and grey literature were excluded. The initial screening was performed by one author and subsequently verified by the second author, with disagreements resolved through discussion. As this was a narrative review rather than a systematic review or meta-analysis, no formal risk-of-bias assessment or systematic study-selection protocol was applied. The purpose of the review was to provide an interpretive synthesis of the available evidence within the defined scope rather than an exhaustive assessment of all published literature.

## 3. Galectins in CRC

### 3.1. Galectin-1

Galectin-1 (Gal-1), a 14 kDa protein containing 135 amino acids encoded by the LGALS1 gene, is one of the most extensively researched galectins [41]. Human Gal-1 is soluble and forms dimers composed of a 22-strand antiparallel β-sandwich maintained by non-covalent binding. Gal-1 is expressed in a wide range of tissues and cell types. It can be found in the cytoplasm and the cell membrane, and it can be secreted into the extracellular matrix (ECM), thus acting both intracellularly and extracellularly [41]. Gal-1 has been demonstrated to play a role in various physiological and pathological processes, such as cell growth, migration, and adhesion. In a general review, not specific to CRC, Gal-1 has also been identified as an important modulator of immune response, including inflammation, tumor progression, immune evasion of cancer cells, and immune tolerance in early pregnancy. Gal-1 has also been implicated in angiogenesis, nervous system development, and muscle differentiation [41]. Functions and molecular mechanisms of Gal-1 in CRC have been extensively studied but need further exploration.

**Gal-1 expression patterns in CRC**

The first work to analyze Gal-1 expression patterns in CRC progression showed that normal epithelial colon cells had negative or weak Gal-1 expression in most cases. By contrast, Gal-1 expression in the stroma significantly increased from normal mucosa to adenomas and carcinomas. In a retrospective single-center cohort of colorectal specimens evaluated by IHC (25 normal mucosae, 15 adenomas, 25 carcinomas, 11 lymph node metastases), stromal Gal-1 overexpression could be observed in 12% of the normal mucosae, 40% of the adenomas, and 84% of the carcinomas [42]. However, no relationship could be established between stromal Gal-1 expression and the grade of dysplasia in the adenomas or the tumor stage, differentiation grade, or size of the carcinomas. As this study is based on a single cohort with a limited sample size, the results should not be interpreted as conclusive without independent validation [42]. Lower expression of Gal-1 mRNA, with decreased protein levels, was observed in persistent and recurrent colorectal adenocarcinomas when compared with non-recurrent cancers [43]. It is worth noting that MALDI-TOF/TOF MS analysis of colorectal adenomas, confirmed by 2-DE, showed the downregulation of Gal-1 in adenomas compared with normal mucosa. This apparent discrepancy likely reflects the study design, as bulk tissue protein content was diluted by Gal-1-negative epithelial cells relative to stroma [44]. In clinical human tissue, Gal-1 was weakly expressed in the endothelial cells (ECs) of normal colon tissue. However, strong expression was found in the ECs of human colon carcinoma using immunohistochemistry (IHC) staining [45]. A comprehensive in vitro RT-PCR study of 22 CRC cell lines detected Gal-1 expression in only 8 of 22 cell lines tested [46].

**Gal-1 in immunity and epithelial–mesenchymal transition (EMT)**

Gal-1 can bind to CD2 and CD3 on human leukemia-derived Jurkat T cell surfaces [47], as well as to CD7, CD43, and CD45 present on the human T cell surface [48], as shown by a study on general human T cell lines and thymocytes. As reviewed by Barrow et al., ligation of CD43/CD45 by Gal-1 leads to caspase-8-mediated apoptosis of T cells [40], a mechanism not yet confirmed in CRC. Extrapolation from these non-CRC specific findings provides hypothesis-generating mechanistic support for Gal-1-mediated immune evasion in CRC.

High tumor Gal-1 expression is associated with reduced eosinophilic infiltration of CRC tissue, which is notable [49], because higher eosinophilic infiltration is considered a favorable condition in CRC [50]. One of the mechanisms by which Gal-1 may contribute to tumor progression involves the modulation of CD8+ regulatory T cell (Treg) activity, thereby attenuating the immune response [51]. A study using the azoxymethane-dextran sodium sulfate model of colitis-associated CRC demonstrated a lower number of tumors and a decreased population of CD8+CD122+PD-1+ Tregs in Gal-1-depleted mice [51]. Silencing of tumor-derived Gal-1 in the CT26 CRC model slowed tumor growth, presumably as a result of the reduced number and inhibited immunosuppressive activity of CD8+CD122+PD-1+ Tregs [51].

In an in vitro direct co-culture of KM12SM cells and bone marrow-derived mesenchymal stem cells (BM-MSCs), Gal-1 expression was upregulated by direct cell–cell contact [52]. This interaction also upregulated other EMT-associated genes, such as fibronectin [52].

Knockout of Gal-1 expression in a teratocarcinoma mouse model attenuated tumor angiogenesis [45]. Moreover, Gal-1 knockdown in an in vitro human umbilical vein endothelial cell (HUVEC) model reduced EC proliferation and migration [45]. These findings may indirectly suggest a similar role of Gal-1 in tumor angiogenesis during CRC progression.

**Molecular mechanisms of Gal-1 in CRC**

**Wnt/β-catenin signaling**

Immunofluorescence staining showed that treatment of KM12C cells with exogenous recombinant human Gal-1 (rhGal-1) induced a cytoplasmic-to-nuclear translocation of β-catenin [53]. Additionally, rhGal-1 strongly induced the expression of the EMT transcription factor Twist1 in KM12C cells [53], which could be suppressed by a β-catenin pathway inhibitor. Secreted Gal-1 was found to have several direct tumor-promoting effects in CRC [53]. Addition of rhGal-1 significantly enhanced the invasive capacity of KM12C cells and decreased expression of E-cadherin at the cell junctions [53]. Sphere formation and drug resistance were both significantly enhanced by rhGal-1 in a dose-dependent manner [53]. The canonical Wnt pathway can be inhibited by 90K glycoprotein, which promotes the degradation of β-catenin via ISGylation-dependent proteasomal ubiquitination [54]. Antitumor effects of 90K were attenuated by overexpression of Gal-1 in CRC cells through a direct interaction at the extracellular region before 90K binds to CD9/CD82 at the cell membrane [54]. On the other hand, circulating 90K was proposed to promote metastasis by aggregating galectin-expressing tumor cells, suggesting that the relative ratio between secreted 90K and galectins in CRC tissues is important for cancer progression and distant metastasis [54]. Further analyses to identify other pathways involved in LGALS1/CTNNB1 interactions demonstrated that SOX9 is an important mediator involved in Gal-1-induced upregulation of β-catenin signaling activity [53].

**Rac1 GTPase pathway**

Rac1 signaling may promote cancer progression by altering critical cellular functions, including the cell cycle, transcription control, and organization of the actin cytoskeleton in SW620 CRC cell line [55]. Rac1 overexpression has been shown to increase the growth of SW620 cells by activating the Wnt signaling pathway and attenuating the TGF-β signaling pathway [55]. Quantitative real-time PCR analysis revealed that Rac1 enhanced the expression of the LGALS1 gene in SW620 cells [55]. Follow-up studies using Western blotting found that Gal-1 expression on the surface of Rac1-depleted cells was significantly decreased [56].

**LYAR-mediated transcriptional regulation**

In the search for novel molecular mechanisms of CRC pathogenesis, the transcription factor LYAR (Ly-1 antibody reactive clone) emerged as a key regulator of the migration and invasion of human CRC cells [57]. LYAR is significantly overexpressed in CRC tissues and correlates with advanced tumor stage and metastasis [57]. Chromatin immunoprecipitation (ChIP) and gene reporter assays have indicated that LYAR directly binds to the LGALS1 promoter, resulting in the activation of Gal-1 gene expression, thereby promoting cell migration and invasion in CRC cells [57].

**CHIP-mediated ubiquitination**

A recent study revealed that the C-terminus of Hsc70-interacting protein (CHIP), a member of the E3 ubiquitin ligase family, can promote Gal-1 ubiquitination and degradation by the proteasome, thereby inhibiting tumor growth and metastasis [58]. Western blotting, IHC staining, and RT-PCR analysis demonstrated significantly higher Gal-1 and lower CHIP expression in CRC tissues compared with the corresponding normal tissues [58]. Moreover, lower CHIP and higher Gal-1 expression correlated with adverse clinical characteristics of tumors and shorter overall patient survival [58].

**Hypoxia-induced Gal-1 expression**

The expression of Gal-1 during tumor development has been shown to be influenced by hypoxia [59]. Ectopically expressed hypoxia-inducible factor 1α protein (HIF-1α) significantly increases Gal-1 expression at both mRNA and protein levels [59]. Silencing of HIF-1α or HIF-1β expression attenuates hypoxia-induced Gal-1 expression, and knockdown of Gal-1 can reduce hypoxia-induced invasion and migration of SW620 cells [59]. This led to a hypothesis that Gal-1 mediated the HIF-1-induced migration and invasion of CRC cells under hypoxic conditions [59].

**TLR4 signaling**

Genetic variations in toll-like receptor 2 (TLR2), TLR3, and TLR4 were shown to be associated with the risk of CRC [60]. In CRC cells stimulated with lipopolysaccharide (LPS), a TLR4 ligand, Gal-1 overexpression contributed to activation of a disintegrin and metalloproteinase domain-containing protein 10 (ADAM10) and ADAM17 [60]. This, in turn, altered glycolytic metabolism and triggered EMT phenotypes [60]. These findings suggest that TLR4-induced Gal-1 expression in cancer cells may promote metastatic and invasive capacity through the modulation of metabolic changes [60].

**Epigenetic modifications of Gal-1**

An analysis of the human LGALS1 promoter revealed that butyrate may upregulate human Gal-1 expression in CRC cells by modulating Sp1 binding to the LGALS1 promoter [61]. Methylation at the CpG-rich sequence at the −614 to −499 bp region in the LGALS1 promoter silenced LGALS1 transcription in Caco-2 and LS180 cells [61]. LS180 cells treated with butyrate displayed de novo biosynthesis of Gal-1 and significantly increased numbers of apoptotic and necrotic cells [61].

**Effects of intracellular Gal-1**

Intracellular expression of Gal-1 in Colo201 cells, as opposed to exogenous Gal-1, induced apoptotic cell death [62]. Intracellular Gal-1 expression also induced apoptosis in LS180 cells, presumably through activation of caspases-3/7 [61]. Moreover, it downregulated the NF-κB signaling pathway through inhibition of IKKα/β and p65 phosphorylation [61]. Gal-1 could also arrest the cell cycle at G0/G1 by decreasing cyclin D1 and increasing p21 expression [61]. This led to the lower proliferation rate, migration and motility of LS180 cells [61]. Overexpression in the intracellular compartment was also associated with decreased β-catenin, TCF-1 and TCF-3 levels [61], suggesting inhibition rather than activation of Wnt signaling.

Taken together, these findings suggest that extracellular Gal-1 is a prominent tumor progression-promoting agent. Extracellular Gal-1 was shown to facilitate cell adhesion dependent on the carbohydrate-recognition domain (CRD) [62]. It may also be involved in key pathological processes such as EMT [52,53], immune suppression [51], and invasion [53]. Conversely, intracellular Gal-1 induces apoptosis [62] and can arrest the cell cycle [61]. This highlights the cell compartment-dependent mechanisms of action rather than a uniformly tumor-promoting or tumor-suppressive function of Gal-1. Evidence on the compartment-dependent role of Gal-1 in CRC is summarized in Table 1.

**Novel Gal-1-modulating agents in CRC**

The apoptotic properties of intracellular Gal-1 are further supported by a study on shikonin, a natural naphthoquinone isolated from *Lithospermum erythrorhizon* [63]. The increased expression of Gal-1 induced by shikonin potently activated apoptosis and autophagic cell death in CRC cells, both in vitro and in vivo [63]. This suggests that shikonin and other Gal-1-inducing agents could represent a novel therapy for CRC [63]. ATP-binding cassette (ABC) transporter B1 (ABCB1) and ABCC1 were shown to be major contributors to multidrug resistance in CRC cells [64]. A novel synthetic acridine-based chalcone (1C) has been recognized as an inhibitor of ABCB1 and ABCC1 [64]. Interestingly, Gal-1 could not be detected in ABCB1-overexpressing Colo320 cells, whereas it was present in Colo205 cells [64]. As opposed to shikonin, compound 1C lowered Gal-1 expression in Colo205 cells, but its anticancer activity was primarily attributed to ABCB1 and ABCC1 inhibition rather than to a simultaneous decrease in Gal-1 levels [64]. Moreover, Gal-1 absence in Colo320 cells suggested a possible yet unknown interaction between Gal-1 and ABCB1 [64].

**Investigating Gal-1 as a biomarker in CRC**

Higher concentrations of Gal-1 in the feces of CRC patients correlated with higher nuclear grade, as well as poor tumor tissue differentiation [65]. Fecal Gal-1 was proposed as a potential non-invasive biomarker of CRC severity [65], but its clinical value must be evaluated against established screening approaches. Besides the well-established fecal immunochemical test [66], a new multitarget fecal immunochemical test [67] and a next-generation multitarget stool DNA test [68] have recently been validated for CRC screening in clinical trials (ClinicalTrials.gov IDs: NCT05314309 and NCT04144738, respectively). Analyses also revealed that fecal Gal-1 significantly correlated with serum AFP and CA 19-9, but not with CEA [65]. Furthermore, no associations were found between serum Gal-1 and AFP, CA 19-9, and CEA [65]. On the other hand, serum CEA negatively correlated with peritumoral tissue expression of Gal-1 [69]. Although these associations are interesting, fecal, serum, and tissue Gal-1 represent different biological compartments and are not directly comparable.

Both Gal-1 and CEA serum concentrations could predict lymph node metastasis (LNM), but the area under the curve (AUC) for CEA was slightly greater than the AUC for Gal-1 (0.638 vs. 0.627) [70]. Gal-1 yielded approximately 77% sensitivity at 40% specificity in this study [70]. An AUC of 0.627 demonstrates relatively weak discrimination, and 40% specificity implies a high false-positive rate. Notably, in patients with normal CEA concentration, Gal-1 could help predict LNM [70].

Serum Gal-1 concentrations were found to be significantly higher in CRC patients with TNM (tumor, node, metastasis) stage III/IV than in those with stage I/II [70]. In another cohort, statistical significance was observed when comparing CRC with liver metastases to healthy controls [71]. However, serum Gal-1 concentrations did not differ in non-metastatic CRC patients compared with healthy controls [71]. No significant differences were found in Gal-1 concentrations in plasma compared with serum [71], but results obtained using different matrices should be interpreted with caution. A study using plasma Gal-1 concentrations set the cut-off value at 340 ng/mL, yielding 90% specificity and 40% sensitivity for CRC detection using sandwich ELISA [72]. These results indicate that Gal-1 assessed by ELISA would not detect most CRC cases, further implying that Gal-1 is a weak univariate marker for CRC.

Elevated plasma Gal-1 levels significantly decreased after tumor-removal surgery [72]. However, no association between serum concentrations of Gal-1 and a 10-year post-surgery survival of patients with CRC was observed [71]. When assessed as an independent factor using IHC-based quantitative computer-assisted microscopy, Gal-1 expression did not provide significantly useful prognostic value for patients across all Dukes stages [73]. However, when only Dukes A and B tumors were analyzed, Gal-1 provided statistically significant survival prediction, where slightly above 80% of patients survived over 10 years [73]. The ability to identify 80% of patients who will survive more than 10 years is notable, but this only applies to early-stage tumors, which is a major limitation of Gal-1 as a standalone predictive marker.

Significantly higher Gal-1, IL-33, and IL-1 concentrations were found in the sera of anemic CRC patients when compared with non-anemic patients [74]. Anemia, as a result of disease severity, positively correlated with lymph and blood vessel invasion, higher TNM stage, and detectable metastatic lesions [74]. This may explain the elevated Gal-1 levels in the sera of anemic patients.

A study to evaluate the diagnostic utility of serum Gal-1 autoantibodies was recently conducted, although statistically significant differences could not be determined when comparing CRC patients with healthy controls [75]. This assay measured host antibodies against Gal-1, which is a fundamentally different approach from Gal-1 expression-based findings described above.

Taken together, current evidence indicates that (regardless of assay method and matrix) Gal-1 as a standalone biomarker for CRC-related endpoints does not provide significant clinical utility. However, further studies are required to evaluate the potential contribution of Gal-1 to a multimarker approach.

### 3.2. Galectin-2

Galectin-2 (Gal-2) is a proto-type galectin and contains only one CRD [76]. However, Gal-2 forms homodimers, which facilitate cell surface receptor ligation [77]. Gal-2 has not been extensively studied in CRC, and its role and molecular mechanisms in this context remain largely unknown. In general biology, the expression of Gal-2 by gastrointestinal epithelial cells is common and contributes to epithelial layer integrity by strengthening the mucus layer [78]. Gal-2 has been implicated in intestinal wound healing and may offer therapeutic benefit in diseases with epithelial barrier disruption, such as inflammatory bowel disease [79]. Moreover, it was shown that Gal-2 may prevent regulatory T cell apoptosis during preeclampsia [80]. Changes in Gal-2 expression patterns have been linked to the pathogenesis of various cancers [71].

**Gal-2 expression patterns in CRC**

Currently, some contradictory reports regarding Gal-2 mRNA expression in CRC and concentrations in the circulating blood of CRC patients can be found. Analysis of plasma samples from 105 CRC patients and 100 healthy volunteers by sandwich ELISA did not reveal any significant difference in Gal-2 concentrations between these two groups [72]. However, another study reported significantly elevated levels of Gal-2 in the serum of both colon and breast cancer patients in comparison to healthy controls [71]. Although this study reported no significant differences in serum compared with plasma [71], the discrepancy in the results may reflect differences in cohort composition between these studies. Interestingly, metastatic cancers were shown to be associated with even higher concentrations of circulating Gal-2 than local tumors [71]. A TCGA and GTEx database search revealed that the relative LGALS2 transcript expression was significantly decreased in colon tumors compared with healthy controls, suggesting that Gal-2 deficiency may be linked to tumorigenesis. The search included 51 normal colon samples and 648 colon tumor samples (Datasets: TCGA CC, GSE14333, GSE8671, and GSE41258); the data were normalized and the tumor origin was clear [81]. Indeed, Gal-2 mRNA expression was lower in tumors from patients with tumor perforation than in those without perforation [43]. Distal CRCs showed a higher prevalence of Gal-2 mRNA downregulation than proximal colon tumors, implying a possible site-dependent role of Gal-2 [43]. However, Gal-2 mRNA expression was higher in early stages of CRC when compared with more advanced stages [43]. Similarly, Gal-2 mRNA overexpression was more prevalent in non-metastatic CRCs and in CRCs without lymphovascular invasion [43]. Some discrepancies within the studies may be explained by the fact that RT-PCR analysis of 22 colorectal cell lines detected only 6 lines that expressed Gal-2 mRNA [46].

**Dual role of Gal-2 in CRC**

A study on Gal-2-knockout mice demonstrated markedly increased tumor size and disrupted epithelial integrity [81]. Activation of STAT3 signaling enhanced proliferation and progression of CRC [82]. Inhibition of STAT3 could induce apoptosis [82]. Western blot analysis demonstrated increased phosphorylation of STAT3 in colon tumors of Gal-2-knockout mice compared with the wild-type mice [81]. In line with these findings, increased Gal-2 expression inhibited the proliferation of HCT116 cells and made them more susceptible to H_2_O_2_ [81]. Taken together, endogenous Gal-2 may have suppressive effects on cancer cell proliferation.

Gal-2 was shown to bind the oncofetal Thomsen-Friedenreich antigen present on the mucin protein MUC1 expressed by colorectal cells [71]. This binding was proposed to contribute to cancer cell adhesion to the vascular endothelium [71]. Additionally, circulating Gal-2 may interact with the vascular endothelial cells in vitro [83]. Upon cell surface receptor ligation by Gal-2, human micro-vascular lung endothelial cells (HMVEC-Ls) secreted G-CSF, IL-6, and GROα [83]. When Gal-2, Gal-4 and Gal-8 were combined, increased adhesion of HCT116 cells to HMVEC-Ls could be observed in a co-culture [83]. It was also demonstrated that these cytokines may induce the expression of cell surface adhesion molecules on HMVEC-Ls in vitro [83]. In a mouse model, combination of these galectins resulted in elevated levels of G-CSF, IL-6, and MCP-1 [83], suggesting a potential role of circulating galectins in cell adhesion and metastasis, but not directly confirmed in CRC by this study. However, the effect of Gal-2 on cytokine release was only observed in vitro. Administration of Gal-2 alone had no effect on cytokine levels in mice [83]. Taken together, circulating Gal-2 may promote metastasis in vitro by facilitating cancer cell adhesion to the vascular endothelium.

**Gal-2 as a CRC survival predictor**

An increase in serum Gal-2 levels was associated with an elevated 10-year mortality risk in CRC patients (18% higher risk with a 2-fold increase in Gal-2 and 75% higher risk with a 10-fold increase) [71]. Although this dose-dependent association was significant (*p* = 0.013) [71], Gal-2 has not been validated as a standalone prognostic marker. Increased or normal Gal-2 mRNA expression in tumor tissue showed a trend toward shorter survival [43]. However, this study yielded a relatively high *p*-value of 0.078 in a cohort of 98 patients with CRC [43]. This might be explained by a limited sample size.

Taken together, experiments revealed anti-tumor effects of endogenous Gal-2 on CRC cells. On the other hand, circulating Gal-2 was shown to potentially promote metastasis in vitro. Although this effect was not confirmed in vivo, the association between elevated serum Gal-2 and increased mortality risk of CRC patients may suggest that circulating Gal-2 contributes to adverse outcomes. However, this remains observational and does not establish causation, since elevated Gal-2 could simply reflect tumor burden. These findings highlight the context-dependent and comparatively underexplored function of Gal-2 in CRC, which warrants further investigation.

### 3.3. Galectin-3

Galectin-3 (Gal-3) is one of the most researched galectins. It is the only chimera-type galectin [84,85] and fails to form dimers [86]. However, it can undergo oligomerization into pentamers to form lattice-like structures upon ligand engagement [87], which is essential for cross-linking cell-surface glycoconjugates, stabilizing receptor clustering, and enhancing downstream signaling [88]. In the context of CRC, the function of Gal-3 and its clinical translation have been extensively studied, but they remain insufficiently validated.

**Gal-3 expression patterns in CRC**

Expression of Gal-3 is common across human tissues and various immune cells [88]. RT-PCR analysis showed that Gal-3 mRNA was present in normal colon cells and in all 22 CRC cell lines tested [46]. Interestingly, in clinical samples, Gal-3 mRNA expression levels varied between rectal cancer (50% high/50% low) and colon cancer (51% high/25% normal/24% low) [43]. At the protein level, IHC-positive staining for Gal-3 was observed in 95% of CRC and 73% of colorectal adenomas [43]. Moreover, it correlated with differentiation status, since 49% of well or moderately differentiated and only 19% of poorly differentiated CRCs showed the highest expression [43]. However, in another cohort, Gal-3 expression was found in 62.5% of cancer tissues but only in 13.0% of cancer-adjacent tissues [89]. IHC also demonstrated that Gal-3 expression in serrated colon lesions, which are precursors of some colon cancers, was significantly upregulated [90]. Abnormal Gal-3 expression appears at early stages of serrated lesions, and the dynamic changes in Gal-3 expression are closely related to their histopathological shift and progression [90]. Gal-3 expression was also shown to be influenced by genomic alterations. It was elevated in microsatellite-stable compared with microsatellite-unstable tumors [91].

A review investigating Gal-3 as a novel biomarker for various pathological conditions and cancers showed compartment-dependent expression and the role of Gal-3 in diseases not related to CRC [92]. For example, Gal-3 was mainly located in the cytoplasm, where it facilitated cell survival by binding to certain survival-associated proteins, such as Bcl-2 [93] and GTP-bound K-Ras [94]. The nuclear fraction of Gal-3 could influence pre-mRNA splicing, leading to altered gene transcription [92]. Extracellular Gal-3, bound to the cell surface or in circulation, was involved in cell–cell and cell–ECM interactions [92].

One of the early studies showed that Gal-3 expression in CRC is downregulated in the initial stages of neoplastic progression, and expression increases in later phases of tumor progression [42]. Nuclear and cytoplasmic Gal-3 levels are significantly downregulated in adenomas [42]. On the other hand, in carcinomas, nuclear Gal-3 shifts to the cytoplasm, resulting in increased cytoplasmic Gal-3 levels [42]. Another study found that Gal-3 was localized in a granulated form in the cytoplasm and on the cell surface of primary CRC cells, but Gal-3 expression levels differed among patients and even within the same section, indicating the multiplicity of Gal-3 expression [95]. In confluent polarized T84 colon carcinoma cells derived from lung metastasis, Gal-3 accumulates at the apical membrane. In subconfluent cells, Gal-3 is localized in proximal parts of lamellipodia [96]. Moreover, evaluation of Gal-3 expression in different tumor regions revealed a significant association with liver metastasis when Gal-3 expression was higher at the surface of a tumor and decreased at the invasive front [97]. The reduction in Gal-3 expression in the primary lesion can be considered a preparatory stage for invasion and metastasis [97]. However, at the site of distant metastasis, the re-expression of Gal-3 was observed [97]. In contrast, the pattern differed at sites of lymph node metastasis [43]. All lymph node metastases with elevated Gal-3 levels showed expression similar to that of their primary tumors but in 80% of Gal-3-negative lymph node metastases, primary tumors overexpressed Gal-3 [43].

**Molecular mechanisms of Gal-3 in CRC**

A bioinformatic analysis using STRING and Cytoscape predicted that the LGALS3-related protein–protein interaction network in CRC cells may be composed of 8 downregulated proteins (SUFU, RUNX2, ELN, MUC2, EGFR, TLR2, KRAS, and MMP2) and 10 upregulated proteins (HRAS, GEMIN4, GSK3B, CCND1, ANXA7, DDOST, LGALS3BP, DMBT1, IL1B, and AXIN1) that interact with Gal-3 [98]. Additionally, MMP9, KDR, DIF, PRKCSH, NRAS, and CDH5 were predicted to interact with Gal-3, but no significant changes in their expression levels were observed [98]. At the protein level, Gal-3 was shown to modulate the expression of its major ligand, MUC2 intestinal mucin in CRC cells [99]. MUC2 levels were directly proportional to Gal-3 expression [99]. Moreover, alterations in the production of Gal-3 and MUC2 have been independently correlated with the malignant behavior of CRC cells [99]. Interestingly, the LGALS3 rs4644 variant was associated with the mucinous component, while the other variant, rs4652, showed no correlation with any clinical characteristics [100].

Gal-3 was found to co-localize with ATP synthase in the mitochondrial inner membrane of CRC cells and to inhibit its enzymatic activity [101]. Consistent with this, intracellular ATP levels showed a modest, although not statistically significant, increase upon Gal-3 knockdown [101]. Additionally, Gal-3 knockdown restored drug-arrested cell-cycle progression in CRC cells, shifting cells arrested at S, S/G2, or G2/M phase by doxorubicin, etoposide, or nocodazole, respectively, back toward G0/G1 [101].

**Wnt/β-catenin signaling**

B-cell receptor-associated protein 31 (BAP31) was demonstrated to be increased in CRC cells [102]. Knockdown of BAP31 substantially reduced cancer stemness, which is linked to the development of chemoresistance in cancers, and reduced the half-maximal inhibitory concentration (IC50) of 5-fluorouracil (5-FU) [102]. Both effects were reversed by BAP31 overexpression. It was suggested that BAP31 may act through Gal-3 inhibition, decreasing the accumulation of β-catenin and leading to the subsequent downregulation of Wnt/β-catenin signaling pathway target genes such as c-MYC or SOX2 [102]. Consistent with this, downregulation of Gal-3 was shown to decrease β-catenin protein levels without changing β-catenin mRNA expression [103]. Furthermore, downregulation of Gal-3 decreased AKT phosphorylation, subsequently increasing GSK-3β activity [103]. This enhanced β-catenin phosphorylation and degradation. Taken together, these findings suggest Gal-3-mediated Wnt signaling regulation by GSK-3β phosphorylation through the PI3K/AKT pathway [103]. Constitutive activation of the Wnt pathway as a result of APC, AXIN1, or CTNNB1 mutations is common in CRC, and Wnt-2 is known to be overexpressed in CRC [104]. Silencing of Wnt-2 signaling at the cell surface or Gal-3 expression inhibited TCF-reporter activity, decreased cytosolic β-catenin levels, and induced apoptosis in CRC cells containing downstream mutations [104]. In addition, upon inhibition of both Wnt-2 and Gal-3, a synergistic effect on Wnt signaling suppression and apoptosis was observed [104]. This may suggest that multiple pathways can regulate Wnt/β-catenin signaling in CRC.

**Gal-3 in cell proliferation, migration, and invasion**

In DLD-1 cells, increased Gal-3 expression was associated with lamellipodia formation and enhanced migration [105]. In the mouse model, Gal-3 correlated with distant lung metastasis [105]. The Gal-3 effect on migration was shown to be mediated specifically by K-Ras, Raf, and ERK1/2 pathways [105].

Exogenous CEA was shown to enhance cell migration even at low concentrations [106]. However, this effect was decreased upon Gal-3 knockdown, without altering CEA expression [106]. IHC demonstrated co-localization of Gal-3 with CEA in tumor tissue [106]. Moreover, Gal-3 could bind to CEA on the cell surface and in the cytoplasm of Caco-2 and DLD-1 cells [106]. Taken together, these may suggest a Gal-3-dependent role of CEA in CRC cell migration.

Human LoVo cell proliferation was shown to be enhanced by adipose-derived (AD) MSC-conditioned media [107]. Replicative senescent MSCs, which secreted more Gal-3, exerted a stronger stimulatory effect than premature AD-MSCs [107]. Gal-3 knockdown in senescent AD-MSCs largely abrogated this MSC-mediated growth-promoting effect on LoVo cells [107]. Exogenous Gal-3 was similarly found to drive cell growth through the MAPK/ERK1/2 pathway [107].

Casein kinase I has been shown to phosphorylate Gal-3, decreasing its binding to laminin and mucin by more than 85% [108]. This effect was completely reversible by protein phosphatase 1 (PP1) in vitro [108]. Notably, the proportion of phosphorylated Gal-3 differed across cell lines [108]. Around 8% of total Gal-3 was phosphorylated in the LS174T CRC cell line with low metastatic potential, but more than 20% in its highly metastatic HM7 and LSLIM6 variants [108]. This raises the hypothesis that phosphatase activity may regulate Gal-3 phosphorylation ratios and influence the malignant potential of CRC cells [108].

The activation of cellular PYK2-GSK3α/β signaling by Gal-3 was identified as one of the mechanisms that stimulates CRC cells to secrete proteases such as cathepsin-B, MMP-1, and MMP-13 [109]. This leads to disrupted epithelial monolayer integrity, elevated permeability, and increased tumor invasiveness [109]. Interestingly, this pathway was found to be necessary specifically for Gal-3-induced cathepsin-B release [109].

Recombinant Gal-3 (rGal-3) enhanced colon cancer cell migration, increased phosphorylation of epidermal growth factor receptor (EGFR), and facilitated the internalization of the EGFR from the cell membrane to the cytoplasm [110]. Knockdown of Gal-3, as well as addition of an EGFR-blocking antibody, decreased colon cancer cell migration [110]. These findings may suggest that Gal-3 promotes migration, to some extent, by EGFR activation and combining Gal-3 inhibition with EGFR-targeting strategies needs further investigation [110].

**Gal-3-mediated angiogenesis, cancer–endothelial cell interactions, and EMT**

In general biology studies, not related to CRC, Gal-3 has been demonstrated to enhance the VEGF-dependent [111,112,113] and VEGF-independent [113] angiogenic response in ECs [111] and HUVECs [111,112,113] in vitro and in mouse models [112,113].

Conventional 5-FU treatment directly attenuated Gal-3 production by HT29 cells in vitro [114]. This would indicate that capecitabine, which metabolizes to 5-FU, should inhibit tumor angiogenesis. However, in a mouse xenograft model using the HT-29 cell line, capecitabine treatment resulted in simultaneous VEGF upregulation, with no difference in microvessel density compared with controls [114]. With concurrent administration of VEGF-inhibiting bevacizumab, a synergistic anti-angiogenic effect emerged [114]. These findings may suggest that this synergy reflected a combined effect of Gal-3 downregulation and VEGF neutralization in capecitabine–bevacizumab combination therapy. However, this study only reported an association between attenuated angiogenesis and Gal-3 downregulation in the combination group and did not establish a direct interaction between Gal-3 and VEGF in CRC [114].

HT29 cell adhesion to HUVECs is significantly greater compared with HT29-5F7 cells [115]. Exogenous Gal-3 did not affect HT29 cells but enhanced adhesion of HT29-5F7 cells, which expressed more MUC1 [115]. An E-selectin-inhibiting antibody attenuated baseline HT29 cell adhesion and Gal-3-induced HT29-5F7 cell adhesion to HUVECs [115]. Neither HT29 cells nor HT29-5F7 cells expressed E-selectin on their cell surfaces, indicating that E-selectin expressed on HUVECs plays a primary role in cell adhesion in this setting [115]. Similar effects were observed for an anti-CD44 antibody [115]. Taken together, these findings suggest that Gal-3 may bind to MUC1 on HT29-5F7 cells, facilitating cell adhesion, and that both CD44 and E-selectin are necessary for Gal-3-mediated cancer-endothelial cell adhesion [115].

Gal-3 was shown to interact with vascular endothelial cells to promote IL-6, G-CSF, sICAM-1, and GM-CSF secretion in vitro and in mouse models [116]. This subsequently enhanced the expression of endothelial cell surface adhesion molecules such as integrin αvβ1, E-selectin, ICAM-1, and VCAM-1 [116]. In patients with metastatic colon cancer, high serum Gal-3 concentrations were associated with increased serum G-CSF, IL-6, and sICAM-1 levels [116]. This may indicate that Gal-3 in the serum of cancer patients increases cancer-endothelial cell adhesion by mediating the release of metastasis-promoting cytokines.

Human platelets, co-cultured with HT29 cells, rapidly adhered to them, increasing COX-2-dependent PGE2 production [117]. This interaction decreased p21WAF1/CIP1 expression and upregulated cyclin B1, resulting in an EMT-associated gene signature [117]. It has been suggested that Gal-3 may bind to platelet collagen receptors, facilitating both transient platelet-cancer cell contact and the release of platelet PDGF. Indeed, an anti-Gal-3 antibody attenuated platelet-induced COX-2 overexpression [117]. These findings suggest that a Gal-3 blockade to prevent platelet-induced tumor-promoting signaling may warrant investigation as a potential therapeutic strategy. However, only cell co-culture was used in the study, and no in vivo therapeutic evidence is available [117].

**Gal-3 and NKp30 interaction**

As one of the principal activating receptors expressed on NK cells, NKp30 triggers cytotoxic activity upon interaction with its ligands, as described in general biology (not CRC-specific) [118]. Ligation of NKp30 drives both NK cell activation and the secretion of immunoregulatory mediators, contributing to host defense against infections and tumors [118]. As a known NKp30 ligand, Gal-3 has been shown to modulate this interaction in CRC [119]. Interestingly, tumor Gal-3 expression was inversely correlated with NKp30 levels on circulating NKT cells [119] (a broadly gated population, not specific to invariant NKT). However, this relationship was reversible. After surgical tumor removal, patients with high tumor Gal-3 expression showed a modest rise in NKp30 on NKT cells [119]. On the other hand, in patients with low pretreatment Gal-3 expression, NKp30 levels declined [119]. This may indicate that Gal-3 is a direct regulator of NKp30 on NKT cells.

**Natural anticancer agents influencing Gal-3**

New agents with potential anticancer activity have recently emerged, including natural compounds such as periplocin, derived from the Chinese herb *Cortex periplocae*. Periplocin alone has been suggested as a potential therapeutic candidate in various cancers, such as papillary thyroid carcinoma [120], pancreatic cancer [121], and non-small-cell lung cancer [122], as well as in esophageal squamous cell carcinoma when combined with TNF-related apoptosis-inducing ligand (TRAIL) [123]. In CRC, it was found to bind Gal-3 and block its Lys210 ubiquitination-mediated proteasomal degradation [124]. This interaction stabilized Gal-3 at sites of periplocin-induced lysosomal damage, subsequently causing excessive lysophagy that led to apoptosis [124]. Periplocin showed anticancer effects in preclinical experimental CRC models, both in vitro and in vivo [124].

Moreover, treatment with Astragalus polysaccharide (APS) was shown to attenuate CRC progression in a murine model by restoring the functional capacity of CD8+ T cells [125]. APS lowered levels of activated STAT3 and Gal-3 within CRC cells [125]. It also downregulated expression of the inhibitory receptor LAG3 found on CD8+ T cells [125]. These findings suggest that the STAT3/Gal-3/LAG3 pathway may warrant investigation as a potential therapeutic strategy [125].

However, to date, no clinical trial investigating periplocin or APS usage in CRC has been conducted. Evidence of periplocin and APS anticancer effects in CRC emerged only from studies using cell lines and short-term a mouse xenograft model. Although periplocin showed no adverse effects on body weight, organ histology or blood biochemistry in the CRC mouse model, oral administration of periplocin in high doses or over long periods caused serious adverse reactions, especially cardiotoxicity [126] and heart injuries in non-CRC rat models [127], which would limit periplocin therapeutic applications. Moreover, periplocin is not a Gal-3-specific agent. In other non-CRC conditions, it was shown to influence multiple signaling pathways, such as DR4-mediated RIPK3 and MLKL signaling [120], AMPK/mTOR pathway [121], or SRC pathway [128], which highlights its broad pharmacological context. Data on APS toxicity are limited, but it has been shown that oral administration of APS-based nanoparticles may be used as a novel drug-delivery strategy to target colon cancer and reduce adverse effects of established anti-tumor agents [129].

**Investigating the clinical relevance of Gal-3 in CRC**

A meta-analysis indicated that in clinical CRC samples, elevated Gal-3 expression assessed by IHC correlated with poor overall survival, advanced TNM and Dukes stages, increased venous invasion, and higher CEA concentration [130]. Gal-3-positive patients had significantly lower median progression-free survival times than Gal-3-negative patients, 19.2 and 35.1 months, respectively [89]. The difference of almost 16 months in disease progression may be clinically significant, but this single-study result requires confirmation. The positive group showed significantly larger tumor sizes, deeper invasion into the colonic wall, and poorer differentiation in histology [131]. The frequency of poorly differentiated adenocarcinomas and mucinous adenocarcinomas reached about 18% in the positive group and only 2% in the negative group [131].

When measured in serum, representing a different biological compartment not directly comparable to tissue expression, Gal-3 concentrations were higher in patients with colon cancer compared to patients with rectal cancer [132]. They were also associated with lower IL-10 and IL-12 production, lower levels of retinol-binding protein, prealbumin, transferrin, and a lower lymphocyte stimulation index, as well as increased IL-17 production, neutrophil/lymphocyte ratio, white blood cell count, and C-reactive protein [132]. Gal-3 concentrations in the serum of patients with CRC and in those with metastases were significantly increased compared with healthy controls, 11.3-fold and 31.6-fold, respectively [71], although this difference itself does not provide clinical utility. Serum Gal-3 levels measured at the time of surgical removal of the primary tumors were not associated with subsequent 10-year survival of CRC patients [133]. Preoperative serum Gal-3 levels were significantly higher in patients with postoperative complications (POC) compared with those in the non-POC group [134]. Additionally, Gal-3 levels increased immediately after surgery in both groups and then normalized, although Gal-3 concentrations in the POC group were significantly higher than those in the non-POC group throughout the perioperative period [134]. When measured one day after resection, serum Gal-3 showed the best predictive performance (AUC = 0.868), with 68.0% sensitivity and 90.0% specificity for POC [134]. The AUC of 0.868 is one of the strongest values provided by a single galectin measurement and may be considered good discrimination. This also indicates that Gal-3 may provide an advantage in correctly distinguishing 90% of patients who will not suffer postoperative complications. However, this result was obtained from a single-center prospective observational study with a small sample size (10 POC and 25 non-POC patients) and must be externally validated on a larger scale.

Interestingly, concentrations of circulating Gal-3 in plasma were found to be even higher than in serum [71], highlighting that serum and plasma are not always interchangeable matrices and should not be treated equally. Gal-3 in plasma, as the only predictor of CRC, yielded a specificity of 43% at 70% sensitivity in univariate analysis [135], indicating poor discriminatory power and limited clinical utility as a standalone biomarker for CRC detection. Additionally, plasma Gal-3 levels were significantly increased in CRC patients compared with high-risk adenoma (HRA) individuals [136]. However, only weak stratification between these 2 groups was obtained by using Gal-3 alone (AUC = 0.59, 19% sensitivity at 90% specificity) [136], which does not support clinical use as a standalone diagnostic tool in this case. Notably, in multivariable analysis, plasma Gal-3 concentration was not included, suggesting that it added no diagnostic value beyond other considered markers [136]. However, simultaneous determination of serum Gal-3 and Gal-4 concentrations showed high specificity and high sensitivity in distinguishing metastatic CRC patients from those without metastases as well as from healthy controls [133]. A cut-off value set at 5 ng/mL resulted in 91% sensitivity and 73% specificity in differentiating patients with and without liver metastases [133]. This finding suggests that Gal-3 may contribute to a multimarker approach in differentiating non-metastatic CRC from metastatic CRC, which is a promising perspective but has not yet been clinically validated.

### 3.4. Galectin-4

Galectin-4 (Gal-4) is a tandem-repeat galectin containing two CRDs [85,137]. Altered expression of Gal-4, particularly at the mRNA level, can be observed in various cancer cell lines, as we noticed in a previous review [138]. Like other galectins, Gal-4 exerts different effects on cell behavior, depending on its cellular location [139]. Compared with Gal-3, the functions and molecular mechanisms of Gal-4 in CRC are less elucidated but remain promising and require further investigation.

**Gal-4 expression patterns and roles in CRC**

Similar to normal mucosal epithelial cells, intracellular Gal-4 was shown to localize mainly adjacent to the basal membrane in confluent T84 cells [96]. In contrast, in subconfluent T84 cells Gal-4 was observed in the leading edge of lamellipodia, at the cell-substrate contact sites in freshly seeded cells, and at the sites of breaks in the monolayer [96]. This indicates that Gal-4 may be involved in cell adhesion and protection of monolayer integrity. Moreover, Gal-4 co-localizes with sulfated glycosphingolipids on the cell surface of CRC cells [140]. When immobilized, Gal-4 binding to sulfated glycosphingolipids was associated with increased adhesion of CCK-81 cells [140]. This interaction suggests that extracellular Gal-4 may facilitate cell–cell adhesion [140]. Gal-4 mRNA was detected by RT-PCR in 14 of 22 CRC cell lines tested, as well as in normal colon tissue [46]. However, Gal-4 mRNA concentrations in tumors obtained from CRC patients were decreased in 18 of the 19 cases [141]. Despite this, no association was found between Gal-4 expression and age, sex, tumor localization, size, or the state of differentiation [141]. Metachronous CRC underexpressed Gal-4 mRNA when compared with non-metachronous CRC [43]. Significantly lower Gal-4 expression levels were also observed by IHC in CRC samples compared with normal colon mucosa tissues [142]. Consistent with this, Gal-4 was demonstrated to be continuously downregulated in the normal-adenoma-carcinoma sequence [143]. Interestingly, exposure of 9 tumor cell lines to sodium butyrate resulted in Gal-4 upregulation in only 3 of them [144]. Further studies revealed the increased proliferation of Gal-4-positive CRC cell lines upon exposure to an anti-Gal-4 antibody, suggesting that the surface-bound Gal-4 functions as a cell growth inhibitor [145]. Furthermore, anti-Gal-4 antibody promoted transcription of inflammatory pathway genes and enhanced secretion of chemokines into the extracellular environment [145]. The above-mentioned observations led to the hypothesis that CRC cells shed Gal-4 expression to produce chemokines that promote their own cell proliferation. However, the underlying proteolytic mechanism of shedding has not been directly demonstrated [145].

**Molecular mechanisms of Gal-4 in CRC**

In a xenograft model, Gal-4 silencing increased cell proliferation and activated NF-κB and STAT3 signaling [142]. Moreover, silencing of Gal-4 led to IL-6 upregulation and promoted the expression of cancer-related genes, such as NF-κB target genes [142]. Gal-4 has been shown to interact with APC, Axin and β-catenin, whereas forced expression of Gal-4 resulted in increased Naked 1 and Ephrin B1 levels, and decreased the levels of β-catenin, Dvl2, TCF1, TCF4, c-Myc, LRP6 and cyclin D1 [146], suggesting that Gal-4 is involved in the regulation of the Wnt signaling pathway. Phosphoproteomic analyses of Gal-4-stimulated LS180 cells demonstrated significantly increased phosphorylation of cofilin-1 at position S3 and more than threefold decreased phosphorylation of the glutamine transporter SLC1A5 at position S503 [147]. Cofilin-1 activity has been hypothesized to link extracellular stimuli to actin cytoskeletal dynamics, affecting cell migration and tumor invasion, whereas deactivation of cofilin-1 by its phosphorylation led to attenuated CRC cell migration [147]. Long-term Gal-4 treatment of CRC cells resulted in a significant decrease in glutamine uptake, although this finding was not directly proven to be influenced by SLC1A5 phosphorylation [147]. Overall, Gal-4 treatment influenced the expression of 11.4% of the quantitated proteins (assessed by the stable isotope labeling by amino acids in cell culture) and indicated downregulation of DNA replication-associated processes, while enhancing secretory and transport functions [148]. Recently, nucleotide polymorphisms in the promoter region of the LGALS4 gene (rs116896264 and rs73933062) were found to be associated with elevated Gal-4 levels [149]. This effect may be a result of insertion or deletion of new binding sites for transcription activators/repressors [149]. Intracellular Gal-4 in CRC cells was suggested to interact with nucleic acid-binding proteins and influence gene transcription [150]. In line with this, Gal-4 upregulated the transcriptional activator protein Pur-beta and MAPKAPK3 [150]. Moreover, Gal-4 induced phosphorylation at three sites (T436, S441 and S445) in FOXK1, at S228 in CK2beta, and particularly at S549 in ZBTB7A with about a 10-fold increase [150]. However, the role of Gal-4-mediated phosphorylation patterns remains unknown [150]. Vitamin D receptor (VDR) expression has been shown to be significantly downregulated in colon cancer and Gal-4 was recognized as a potential VDR target gene, providing additional information about Gal-4 regulation [151].

**Investigating the clinical relevance of Gal-4**

Bioinformatic analysis of microarray datasets suggested an association between downregulation of the LGALS4 gene in CRC cells, among many other dysregulated genes, and acquired oxaliplatin resistance, which is the primary cause of CRC treatment failure [152]. Patients with higher levels of tumor tissue Gal-4 mRNA had slightly better survival rates [43]. When assessed by IHC staining, which is a different methodology, not directly comparable to bulk tissue mRNA measurement, higher Gal-4 expression was associated with unfavorable prognosis [73]. The best prognostic values were obtained by combining the expression levels of Gal-1 and Gal-4 [73]. The combined model predicted that almost 100% of Gal-1-negative and Gal-4-negative patients survived 10 years, whereas 0% of Gal-1-positive and Gal-4-positive patients survived more than 6 years [73]. However, this prediction was only statistically significant in Dukes A and B stages and did not provide any prognostic value for patients in Dukes C and D stages [73]. This model provides almost perfect stratification of patients who will survive more than 10 years, which is promising, but it is significantly limited to early-stage tumors and would require confirmation.

In contrast to Gal-4 tissue expression, the concentrations of circulating Gal-4 have been shown to be increased in numerous studies using different methods [71,72,153], but results obtained from different assays and matrices should not be interpreted as interchangeable. This might reflect the previously proposed hypothesis of active shedding or secretion of Gal-4 from tumor cells, although it remains unconfirmed. The evaluation of Gal-4 expression as a biomarker using RT-qPCR whole-blood screening test to detect CRC presence resulted in an AUC of 0.746 with 61.2% specificity and 82.1% sensitivity [153]. However, when combined with TSPAN8, specificity and sensitivity increased to 67.16% and 92.5%, respectively [153]. These results indicate that Gal-4 mRNA, as a univariate marker, not only provides moderate discrimination but may also provide additional diagnostic utility in a multimarker approach. Sandwich ELISA for CRC detection with the cut-off value of plasma Gal-4 set at 0.525 ng/mL yielded 90.0% specificity and 48.6% sensitivity for CRC detection [72]. This indicates that ELISA-determined Gal-4 at the protein level fails to detect over half of CRC cases, but only 10% of patients without CRC would have to undergo unnecessary confirmatory testing. Elevated concentrations of serum Gal-4 in patients with metastasis were observed when compared to patients with non-metastatic CRC, although without significant association with patients’ survival [71]. Gal-4 is also regarded as one of several candidate prognostic biomarkers in a multi-biomarker approach [154,155,156,157], but it requires clinical validation in larger multi-center cohorts.

### 3.5. Galectin-7

Galectin-7 (Gal-7) is a 14 kDa proto-type galectin that is commonly expressed in stratified epithelia, where it is associated with differentiation, development, and migration of epithelial cells [158]. It was also shown to contribute to wound healing [159]. The role of Gal-7 in cancer is dependent on the origin of the tumor and can either be suppressive or promotive [159]. For example, Gal-7 has been suggested to induce MMP-9 expression through the p38 MAPK pathway in cervical cancer HeLa cells [160], leading to progression and metastasis. On the other hand, Gal-7 inhibited neuroblastoma cell proliferation [161]. Although several studies have investigated the role of Gal-7 in CRC, the evidence remains rather limited. This highlights that Gal-7 is a comparatively underexplored galectin in CRC and warrants further investigation.

**Gal-7 expression patterns in CRC**

Gal-7 expression by CRC cells seems to be a common feature, since only 6 out of 22 CRC cell lines did not express Gal-7 mRNA in RT-PCR analysis [46]. Gal-7 mRNA could also be detected in normal colon tissue [46]. Interestingly, azoxymethane induction of rat colon adenocarcinomas resulted in tumors where no detectable signal of Gal-7 expression could be obtained by Northern blot analysis [162]. The surface presence of Gal-7 on the Rho GTPase Rac1-depleted SW620 cell line was barely detectable [56]. Analysis of the protein level with non-cross-reactive antibodies against Gal-7 in NaBut-treated CRC cell lines revealed that Gal-7 was upregulated in five out of nine lines [144]. However, since the effect depended on the cell line, p53 is rather uninvolved [144]. DLD-1 cells did not express Gal-7 mRNA [46,163].

**Mechanistic and functional role of Gal-7 in CRC**

Interestingly, upon the introduction of Gal-7 ectopic expression to DLD-1 cells, a sensitization to apoptotic stimuli could be observed [163,164]. After actinomycin D treatment, Gal-7-transfected DLD-1 cells (DLD-1-Gal-7) showed an elevated percentage of annexin V-positive cells, corresponding to cells in the early stage of apoptosis [163]. This finding was later corroborated by another study and extended to hypoxic and low-serum conditions [164]. DLD-1-Gal-7 transfectants grew significantly more slowly and formed significantly lower numbers of colonies than control transfectants in vitro [164]. When subcutaneously injected into severe combined immunodeficient mice, the proliferation rate of DLD-1-Gal-7 cells was dramatically reduced, thus slowing tumor formation [164]. However, caspase-3 activity in vivo was equally low in transfectants and controls, indicating that the attenuated tumor growth rate is not an effect of apoptosis [164]. This may also be explained by the fact that DLD-1-Gal-7 tumors contained a lower density of blood vessels, suggesting a potential role of Gal-7 in angiogenesis suppression in vivo [164]. The mechanism by which Gal-7 may influence angiogenesis was not determined and remains unexplored.

Euchromatic histone lysine methyltransferase 2 (EHMT2) is a known epigenetic repressor that, together with chromodomain helicase DNA-binding protein 4 (CHD4), reduces chromatin accessibility and suppresses Gal-7 expression [165]. A recent study suggested that the EHMT2/Gal-7 axis may facilitate the recruitment and activation of CD8+ T cells in microsatellite-stable (MSS) tumors [165]. However, this suggestion is based on the observation rather than experimental determination of direct receptor–ligand interaction, indicating that the potential molecular mechanism underlying Gal-7 interaction with CD8+ T cells needs further elucidation.

**Investigating the clinical relevance of Gal-7 in CRC**

An observational study revealed that Gal-7 mRNA was frequently overexpressed in early-stage CRC tissues when compared with advanced stages [43]. Moreover, Gal-7 overexpression was noted in 100% of carcinomas without lymph node metastasis, as opposed to 82% of carcinomas with lymph node metastasis [43]. Gal-7 mRNA expression was also lower in patients with tumor perforation when compared with those without perforation [43]. Despite this, in a study analyzing galectins in colon carcinoma tissue samples obtained from patients treated with standard therapy, a prognostic value could not be established for Gal-7 [73]. Moreover, plasma concentrations of Gal-7 in the CRC patient group compared with healthy controls showed no significant difference [72]. Conversely, another study on CRC patients showed that Gal-7 was downregulated in the serum of patients with early CRC, and at the same time, CRC tissues showed 100% negative immunoreactivity to Gal-7 [166]. This underscores the promising but not yet established clinical relevance of Gal-7 in CRC.

### 3.6. Galectin-8

Galectin-8 (Gal-8) is involved in cell proliferation, cell adhesion, apoptosis, and immunity [167]. In a broad non-CRC-specific context, Gal-8 has been shown to interact with MRC2/uPAR/LRP1 [168], integrins [169], CD44 [170], and CD166 [171]. As reviewed in an article about Gal-8 in general biology, it can activate FAK, ERK, AKT, and JNK signaling pathways [167], leading to cytokine and chemokine release. Moreover, Gal-8 can bind to ECM proteins and was shown to exert some contradictory effects on cell adhesion in Chinese hamster ovary (CHO-P) cell culture [172]. For example, binding of Gal-8 to the cell surface inhibits cell adhesion [172], indicating Gal-8 affinity for receptors mediating cell–matrix interactions. Conversely, when immobilized onto the matrix, Gal-8 facilitates cell adhesion of CHO-P cells [172], as well as migration of CHO-P cells [172] and human ECs [173]. The biological function of Gal-8 in CRC is intriguing, but its clinical significance remains inconclusive.

**Gal-8 expression patterns in CRC**

Studies show that Gal-8 is commonly expressed among various cancer cell lines. RT-PCR showed Gal-8 expression in all 22 tested CRC cell lines [46]. It was shown that Gal-8 can bind to human CRC cells, attenuating HCT15 and Colo201 cell migration [174]. However, faster growing LoVo and DLD-1 cells were not affected, which suggests that sensitivity to Gal-8 is associated with cell growth rate. Gal-8 mRNA measured by RT-PCR, as well as corresponding protein assessed by IHC, has been detected in all four above-mentioned cell lines [174]. Upon introduction into nude mice, both Gal-8 levels and the amount of immunopositive epithelial cells inversely correlated with the tumor growth rate [174]. Similarly, in more aggressive CRC lines, such as SKCO1 and HCT116, Western blot showed lower Gal-8 expression than in the other less aggressive CRC cells [175]. In another in vitro study using RT-PCR, HT29 and HCT116 showed high expression of Gal-8 [176]. However, immunoblotting failed to detect Gal-8 protein in cell lysates [176]. Notably, HCT15, LoVo, Colo201, and DLD-1 showed more intense Gal-8-dependent staining in vitro than in vivo [174]. RT-qPCR analysis found elevated expression of Gal-8 in CRC tissue with slight reduction in Gal-8 overexpression in more malignant cases [43]. IHC-determined expression levels of Gal-8 show significant variation in vivo [177]. These results may appear discrepant, but RT-PCR and IHC consistently reported elevated Gal-8 expression in more malignant cell lines, whereas Western blot failed to detect or report decreased Gal-8 at the protein level. This highlights the importance of appropriate method selection and underscores that results obtained from different assays should not be interpreted as interchangeable. Weaker expression of Gal-8 in vivo than in vitro may be explained by complex interactions between cancer cells and tumor microenvironment.

Consistent with the above-mentioned weaker staining in vivo than in vitro [174], in pair-wise IHC staining of human CRC tissues, approximately 36% of tumors did not overexpress Gal-8, about 19% showed weak expression, and strong expression was observed only in 17% of tumors [177]. Moreover, expression of Gal-8 increased from normal to benign stages and decreased in locally invasive and extensively invasive carcinomas [174]. Similarly, CRC samples showed that Gal-8 expression is downregulated during CRC tumor progression, with a significant difference between stages T1 and T4 [175]. In normal and benign colon tissue, Gal-8 is present in the cytoplasm and the nuclei [174]. However, in malignant colon cells, Gal-8 is no longer detectable in the nuclei and shifts into the cytoplasm [174].

Additionally, serum concentrations were elevated in patients with CRC, with further significant increase associated with metastasis [71]. These expression patterns are similar to that of Gal-4 and may suggest that reduced expression of Gal-8 in cancer cells or its increased secretion from cancer cells is favorable for cancer progression.

**Molecular mechanisms of Gal-8 in CRC**

In a mouse model, enhanced Gal-8 expression upon CRC introduction attenuated tumor growth and reduced liver metastasis [175]. Simultaneous downregulation of MMP-2 mRNA levels was observed [175]. Moreover, increased Gal-8 expression correlated with reduced phosphorylation of Src, decreased vimentin levels, and elevated E-cadherin levels [175]. Gal-8-knockdown reduced E-cadherin levels, upregulated EMT-protein expression (including vimentin), and increased Src and RAS activation [175]. Gal-8-transfected MC38 cells injected into C57BL/6J mice formed tumors that grew faster than the wild-type tumors [177]. This indicates that Gal-8 may promote tumor growth and progression in vivo. Moreover, Gal-8 increased FoxP3-positive regulatory T cell count and decreased CD8+ T cell infiltration in MC38 tumors [177]. The apparent discrepancy between these two studies arises from different model designs. The syngeneic MC38 model was fully immunocompetent and revealed that Gal-8 may influence the tumor microenvironment to induce immune suppression and enhance tumor growth [177]. On the other hand, the xenograft immunodeficient mouse model lacked the interaction between Gal-8 and T cells, only revealing the effect of Gal-8-mediated TGF-β signaling inhibition in cancer cells.

Mutations of TGFBR2 (encoding the type II TGF-β receptor-TβRII) or TGFBR1, impairing TGF-β signaling, can be found in CRC. Gal-8 was shown to co-localize with TβRII on HT29 cells, and the binding of Gal-8 to TβRII attenuated cell migration, regardless of TGFBR1/2 mutations [175,178]. Recombinant Gal-8 (rGal-8) administration induced the activation of caspases and temporal activation of JNK and ERK in DLD-1 and HT29 cells, leading to apoptosis [175]. Furthermore, rGal-8 reduced malignant properties of CRC cells, such as cell migration [175]. β4-Galactosyltransferase-I (B4GALT1) knockdown effectively attenuated the metastasis-inhibiting effects of Gal-8 and substantially reduced Gal-8 binding to TβRII [175]. Interestingly, rGal-8 had no effect on normal human colorectal epithelial cells [175].

Gal-8, similar to other galectin family members, can bind to TF antigen and promote CRC cell adhesion to vascular endothelium [71]. Rho GTPase Rac1-depleted SW620 cells showed increased cell surface localization of Gal-8 [56].

**Gal-8 and prognosis in CRC**

MSI-H tumors with high mutation burden were shown to overexpress Gal-8 at the transcript level [177]. Moreover, in the MSI-L/MSS tumors, Gal-8 further increased with tumor progression [177]. This study also reported a negative role of Gal-8 in the prognosis of CRC. Higher expression was significantly associated with shorter survival time (*p* = 0.011) [177]. However, this association remains a single study finding that requires independent validation. Gal-8 also provided a two-group stratification with a significant prognostic value [73]. However, classification based on Dukes stages C and D performed better [73].

Serum Gal-8 concentrations in CRC patients showed a modest 1.8-fold increase when compared with healthy controls [71]. Moreover, in metastatic cancer, Gal-8 concentrations increased 5.6-fold when compared with healthy controls [71]. Despite that, no correlation with patients’ survival could be established [71]. This correlation of elevated serum Gal-8 in patients with metastasis was shown in an early study that required contemporary validation using standardized serum or plasma assays.

Taken together, Gal-8 currently does not possess any established prognostic value, although studies investigating possible Gal-8 clinical significance are very limited.

### 3.7. Galectin-9

Galectin-9 (Gal-9), encoded by the LGALS9 gene, is a tandem-repeat galectin with two CRDs connected by a linker domain [179,180]. Interestingly, as discussed by Aanhane et al., alternative splicing can produce different Gal-9 isoforms such as Gal-9N and Gal-9C that are composed of a single CRD, or long, medium, and short isoforms depending on the linker-peptide size (Gal-9L/Gal-9M/Gal-9S) [181]. Gal-9 has been found in the nuclei of colonic endothelial cells [182] and metastatic melanoma cells [183]. It is most likely secreted by a non-classical secretory pathway, since it lacks the endoplasmic reticulum transport signal sequence [179]. Cell surface Gal-9 was involved in cell aggregation and apoptosis of melanoma cells [183]. Moreover, in general experiments using immune cells, extracellular Gal-9 was shown to function as an ECM protein that binds to cell surface receptors such as T cell immunoglobulin and mucin-domain-containing protein-3 (TIM-3) [184] and Protein Disulphide Isomerase (PDI) [185]. A review of Gal-9 as a biomarker for severity of many diseases showed Gal-9 involvement in chemoattraction, cell adhesion, cell migration, and receptor endocytosis [186]. The role of Gal-9 in CRC has been extensively studied, but its clinical translation requires further investigation.

**Gal-9 expression patterns in CRC**

Expression of Gal-9 mRNA is differentially regulated in specific organs [186], and in the colon it tends to be high [187]. The transcript representing the expected Gal-9 fragment could be detected by RT-PCR in 13 of 22 CRC cell lines [46]. However, the transcript representing a Gal-9 isoform could be detected in 14 cell lines, of which 3 were negative for the expected Gal-9 fragment. Both the expected Gal-9 fragment and the isoform were absent in normal human colon cells [46]. Moreover, decreased protein levels of Gal-9 with lowered surface availability were detected in Rac1-knockdown SW620 cells [56]. Although Gal-9 localizes in nuclei of normal colonic endothelial cells, in CRC tissue a shift in Gal-9 to the cytoplasm of endothelial cells could be observed [182]. IHC staining showed high expression of TIM-3 in 70.18% of CRC tissues from radical resection [188]. However, high expression of Gal-9 was observed in only 32.16% of CRC tissues [188]. Patients with high TIM-3 expression had significantly lower relapse-free survival (RFS) and overall survival (OS) than those with low expression [188]. Conversely, patients with low Gal-9 expression had significantly lower RFS and OS than those with high expression [188], which appears to oppose general ligand–receptor pair interactions, but this may reflect a compartment-dependent or isoform-specific role of Gal-9. IHC-assessed Gal-9 expression in CRC tissue was also lower in another study [189]. The expression levels of Gal-9 were decreased in mismatch repair-proficient patients compared with mismatch repair-deficient patients (bioinformatic mRNA analysis and IHC) [190]. However, ELISA-determined TIM-3 and Gal-9 protein levels yielded completely different results. Both proteins were significantly increased in CRC tissues compared with matched non-tumor margins [191]. Using a qPCR-based fold-change classification (>2-fold relative to matched non-cancerous tissue was considered high expression), high Gal-9 mRNA expression was detected in almost 88 of 98 (89.8%) [43] of CRC tissues obtained from resection surgery.

These apparently discrepant results between the study that reported almost 90% of CRC tissues with high Gal-9 expression, as assessed by qPCR, and other studies that suggest reduced Gal-9 expression may be explained by several factors. First and foremost, these studies used different measurement platforms, measuring expression at different levels: bulk mRNA, fixed-tissue IHC, or homogenate protein. These results may also reflect specific characteristics of a particular cohort rather than a universal expression pattern. Furthermore, differences in tumor stage, molecular subtype, immune-cell infiltration, and Gal-9 isoform composition may contribute to the expression heterogeneity.

**Molecular mechanisms of Gal-9 in CRC**

**Gal-9 in cell proliferation, migration, and apoptosis**

The LoVo cell line has been shown to express E-selectin, as opposed to HCT116 and SW1116 cells [192]. Ectopic expression of Gal-9L decreased E-selectin mRNA levels in LoVo cells, whereas Gal-9M and Gal-9S increased E-selectin mRNA expression, concurrently increasing adhesion to endothelial cells [192]. Treatment with an anti-Gal-9 antibody, β-lactose, or an anti-E-selectin antibody attenuated adhesion capability, indicating that both Gal-9 and E-selectin are involved in the adhesion of LoVo cells to the endothelium [192]. In vitro, Gal-9 only slightly suppressed the proliferation of Colon26 cells in a dose-dependent manner and did not induce cell death [193]. However, in a mouse model, intravenous Gal-9 administration reduced the number of lung metastases of Colon26 cells, which express CD44 [193]. Gal-9 has been shown to suppress the binding of hyaluronic acid to CD44, suggesting one of the potential mechanisms of tumor migration suppression [193]. Moreover, treatment of colon cancer cells with Gal-9 increased caspase-cleaved keratin 18 (CCK-18) levels, a marker of apoptosis [194]. It also markedly altered microRNA (miR-1246, miR-15b-5p, and miR-1237) expression and enhanced the phosphorylation of ALK, DDR1, and EphA10 proteins [194]. CCK-18 increase was observed in Caco-2 and CW-2 cell lines, which are sensitive to Gal-9, but not in the Gal-9-resistant WiDr cell line [194]. Deubiquitinase ATXN3 has recently been recognized as an anti-tumor protein amplifying Gal-9-induced apoptosis of colon cancer cells by protecting Gal-9 from proteasomal degradation [195]. ATXN3 deletion intensified the growth of HCT116 and MC38 cell lines both in vitro and in xenograft models [195]. Ectopic expression of Gal-9 fully reversed the growth of ATXN3-depleted colon cancer in mice [195].

**Tumor microenvironment immune regulation may be mediated by Gal-9**

A strong positive correlation between LGALS9 and CD137 expression has been reported [196]. In mouse models with CD137 overexpression, Gal-9 administration increased the infiltration of activated T cells, promoting anti-tumor activity [196]. In CD137-deficient models, Gal-9 stimulation led to enhanced tumor growth with limited T cell presence, suggesting the CD137-expression-dependent role of LGALS9 in CRC [196]. Moreover, increased Gal-9 expression was significantly associated with enhanced infiltration of mature CD208+ DCs, CD8+ T cells, and CD3+ T cells, whereas attenuated infiltration of immature CD1a+ DCs could be observed [190]. A linear correlation between Gal-9 expression and CD56+ NK cell infiltration into CRC tissue has been demonstrated [189]. Gal-9 stimulated migration of human NK-92 cells by affecting F-actin polarization through the Rho/ROCK1 signaling pathway [189]. Gal-9 expression was elevated in DCs and macrophages in CRC, as was the level of HAVCR2 in NK cells [197]. Recently, the LGALS9-HAVCR2 axis was identified as one of the CRC-specific pathways, enabling communication between NK cells and myeloid cells under CRC conditions [197]. HAVCR2+ NK cells attenuated NK cell-mediated cytotoxicity in CRC [197], indicating an increase in immune evasion.

**The role of the Gal-9/TIM-3 axis in CRC**

Indirect evidence from hepatocellular carcinoma (HCC) indicated that the expression of CD25, FoxP3, CTLA-4 and GITR was enhanced in TIM-3+ CD4+ T cells when compared with TIM-3- CD4+ T cells, and they also produced less IFN-γ and IL-2 [198]. These TIM-3+ CD4+ T cells in HCC were shown to inhibit CD8+ T cell proliferation in vitro. Similar TIM-3+ CD4+ T cells with attenuated production of IFN-γ and IL-2 were found in CRC [198]. The population of tumor-infiltrating TIM-3+ CD4+ T cells was significantly greater compared with corresponding normal colon tissue [198]. However, no suppression assay was performed in CRC, and the extrapolation that TIM-3+ CD4+ T cells in CRC may have played a role in immune suppression in the tumor microenvironment remains a hypothesis. This study also found a direct interaction between TIM-3+ CD4+ T cells and Gal-9+ cells in HCC, suggesting that TIM-3 and Gal-9 can bind in vivo [198].

In CRC, TIM-3 and Gal-9 levels correlated with key immunomodulatory pathways, including IL-10, IL-17, chemokine signaling, upregulation of the cell cycle, as well as downregulation of the interferon response and the TNF-α/NF-κB pathway [191]. These findings may indicate that TIM-3 and Gal-9 expression are hallmarks of cell proliferation and facilitate tumor immune evasion.

TIM-3 levels were significantly elevated in PIK3CA-mutated tumors [191]. However, no associations between TIM-3 or Gal-9 and KRAS, NRAS, BRAF, AKT1, or MSI status were found [191]. Binding of both H3K9me3 and H3K27me3 repressive histones to the TIM-3 promoter region was significantly lower in tumor tissue compared with normal tissue [199], indicating that epigenetic modifications other than DNA hypomethylation may be involved in upregulation of TIM-3. Although Gal-9 and TIM-3 expression were found significantly higher in CRC, neither DNA hypomethylation nor repressive histone binding was significantly associated with Gal-9 expression [199].

Gal-9 interaction with TIM-3 and its influence on immune cells, including CD8+ T cells, CD4+ T cells, and macrophages, have been extensively studied. Interestingly, the population of tissue-resident TIM-3+ CD8+ T cells is larger in CRC tissue than in peripheral blood [200]. In the mouse CT26 colon tumor model, Gal-9 secreted by tumor cells was shown to increase the apoptosis of tumor-infiltrating CD8+ T cells, the majority of which express TIM-3 [200]. Additionally, TIM-3+ cells exhibited elevated cytolytic activity, effector cytokine secretion and apoptosis compared with the TIM-3- population [200]. Furthermore, anti-TIM-3 antibody treatment reduced apoptosis and inhibited tumor growth, concurrently increasing the therapeutic efficacy of cyclophosphamide in mice [200]. Conversely, a rat anti-mouse Gal-9 monoclonal antibody caused only moderate and transient anti-tumor effects in the MC-38 mouse colon cancer model [201], implicating other important interactions between Gal-9 and the tumor microenvironment.

Further studies revealed that the TIM-3/Gal-9 axis is involved in p85 binding to the cytoplasmic domain of TIM-3 [202]. The binding of p85 to TIM-3 reduced available p85 that could interact with PI3K-p110 [202]. This attenuated PI3K/AKT signaling and resulted in the polarization of M0 macrophages towards the M2 phenotype [202]. M2 macrophages were shown to enhance tumor growth and facilitate metastasis [202]. Interestingly, the polarization was reduced by anti-TIM-3 antibodies even more than by Gal-9 inhibition, suggesting that TIM-3 may have multiple ligands [202]. Pro-apoptotic mitochondrial dysfunction decreases Gal-9 translation by reducing Gal-9 mRNA transcription [203]. Pharmacologically induced loss of mitochondrial function reduces Gal-9 exocytosis and causes redistribution of the TIM-3-Gal-9 complex to the mitochondria in CRC cells, where Gal-9 could possibly interact with mitochondrial glycoproteins [203]. The role of this process is yet to be determined, but the transfer of Gal-9 into mitochondria may prohibit the protection of a dying cell [203].

**Circulating Gal-9 may predict CRC in elderly patients**

A recent study recognized a combination of soluble Gal-9, IL-10 and CXCL10 as a promising multi-biomarker approach for CRC detection in elderly individuals, demonstrating an AUC value of 1.000 (95% confidence interval: 1.000–1.000) and effectively stratifying elderly healthy volunteers (HVs) and CRC patients [204]. However, this study used a backward stepwise logistic regression for selecting this three-biomarker model out of 24 variables and calculated the AUC on the same sample, leading to optimistic bias. This result requires cautious interpretation and validation in an independent cohort.

Another finding in this study showed that plasma levels of Gal-9 and sTIM-3 were significantly lower in middle-aged HVs compared with middle-aged CRC patients, and in elderly HVs compared with elderly CRC patients. Interestingly, plasma levels of Gal-9 and sTIM-3 were significantly higher in middle-aged HVs compared with elderly HVs [204]. When measured in serum, a different matrix, Gal-9 concentrations were significantly elevated in patients with metastatic colon cancer [71].

**Gal-9 and therapy strategies in CRC**

A bioinformatic analysis using mRNA expression data from the Affymetrix Human Genome U133 Plus 2.0 Array suggested that the antibody-dependent cell-mediated cytotoxicity (ADCC) strategy alone is unlikely to succeed in CRC [205]. Single-cell transcriptomic analyses of colorectal liver metastases (CRLMs) revealed that about 56% expressed Gal-9 [206]. CRLMs with an enterocyte phenotype were associated with TIM-3 ligand expression and exhibited a synergistic response to chemotherapy when combined with TIM-3 blockade [206]. Afatinib is an irreversible EGFR tyrosine kinase inhibitor (TKI). In a syngeneic CT26 colon tumor model, Gal-9 inhibition enhanced the response of EGFRwt tumors to afatinib [207]. The suggested mechanism of action involved DC-mediated T cell priming, leading to enhanced infiltration of T cells into tumors [207]. CRC with an oncogenic KRAS mutation (KRASmut) is resistant to both chemotherapy and EGFR-targeted therapy [208]. However, KRASmut CRC has been shown to be sensitive to treatment with recombinant Gal-9, which acts as a lysosomal inhibitor of autophagosome–lysosome fusion, leading to autophagosome accumulation, excessive lysosomal swelling, and cell death [209]. In human cancer cells, hypoxia-inducible factor 1 (HIF-1) and activator protein 1 (AP-1) are involved in the upregulation of TGF-β1 expression, which activates the transcription factor SMAD3 through autocrine action, subsequently increasing Gal-9 expression in malignant CRC cells [210]. This relationship was not observed in mature non-transformed cells, suggesting the importance of the TGF-β1 signaling pathway in cancer immunotherapy [210].

**Dual role of Gal-9 in CRC**

Gal-9, similarly to Gal-2, appears to play contradictory roles in CRC depending on its cellular compartment, receptor availability, and isoform. It is helpful to distinguish between TIM-3-dependent and TIM-3-independent effects of Gal-9, since they engage different pathways and cell populations. Acting independently of TIM-3, Gal-9L modulates E-selectin-dependent adhesion [192]. On the other hand, Gal-9M and Gal-9S had opposing effects, highlighting isoform-specific interactions in vitro [192]. Although the specific mechanism underlying these opposing effects needs further investigation, it has been shown that ectopic expression and recombinant Gal-9 may play a pro-apoptotic and anti-metastatic role in CRC [192,193,194,195,209]. The induction of lysosomal cell death in KRASmut CRC cells [209], caspase-dependent apoptosis [194], and inhibition of CD44-mediated cell migration [193] were all cancer cell-related responses.

By contrast, the immunosuppressive effects of Gal-9 were particularly shown to be mediated by the TIM-3/Gal-9 axis through TIM-3-dependent interactions with immune cells [197,200,202]. This highlights the compartment- and immune composition-dependent role of Gal-9. TIM-3-dependent interaction was involved in apoptosis of tumor-infiltrating CD8+ T cells [200], in macrophage polarization toward tumor-promoting M2 phenotype [202], and in the presence of potentially suppressive TIM-3+ CD4+ T cell population [198]. Gal-9 also attenuated NK cell-mediated cytotoxicity [197]. Taken together, this may indicate that Gal-9 interaction with immune cells facilitates immune evasion in the tumor microenvironment.

Although the role of Gal-9 in CRC has been extensively studied, its potential as a biomarker remains to be fully explored, since data on its clinical relevance are limited. The potential of Gal-9 as a therapy target requires further investigation and clinical validation.

### 3.8. Galectin-10

Galectin-10 (Gal-10), encoded by the CLC gene, is expressed in great amounts by human eosinophils [211] and to a lesser extent in basophils [212]. In contrast to the well-characterized role of Gal-10 in eosinophil-related diseases, the role of Gal-10 in CRC is emerging and largely speculative. It has been extensively researched in asthma, eosinophilic esophagitis, rhinitis, sinusitis, and atopic dermatitis [213]. Gal-10 is the main protein component of Charcot–Leyden crystals (CLCs), which are generated upon eosinophil extracellular trap cell death (EETosis), and is not released through conventional secretory processes such as degranulation or exocytosis [213]. It is known that solid tumors can be infiltrated by eosinophils, and the level of CLC protein correlates with eosinophil density in inflammation [214]. CLCs can also be found in macrophages and some T cell subpopulations [214]. For example, in the stroma of mastocytomas, where eosinophils are present, CLCs can be found in macrophages, potentially as a result of phagocytosis [215]. The overall role of eosinophils in tumors has been recognized [216]. Apart from CLCs, eosinophils affect tumors through interferon regulatory factor 5 (IRF5) signaling, which triggers the secretion of several pro-inflammatory cytokines, including IL-1α, IL-1β, and TNF-α [217]. Moreover, IRF5-activated eosinophils recruit and activate CD8+ T cells, amplifying the anti-tumoral response [217].

**Gal-10 expression patterns in CRC**

Observational studies report that CLC was the most differentially expressed gene in early-onset compared with late-onset colorectal tumors, with a 10-fold higher expression [218]. Gal-10 immunostaining was found negative in normal colonic mucosa (NC) and expression of Gal-10 increased in all pathological stages of CRC, in adenoma (AD), carcinoma in situ (CIS), and invasive CRC (ICC) [219]. However, the expression level was lower at CIS than at AD or ICC [219]. These results should not be interpreted as evidence that tumor cells themselves produce Gal-10, since no data about eosinophil density, eosinophil activation or extracellular deposition of Gal-10 through EETosis were reported. It is plausible that one or more of these factors are reflected in the pattern. However, tumor-infiltrating eosinophil density has been reported to change dramatically in a compartment-dependent manner [220]. It was comparable in AD, CIS and on the invasive front of ICC, but it was markedly decreased in ICC stroma [220]. This highlights the need to separate samples based on their spatial origin. To further elucidate Gal-10 expression patterns, co-staining using an eosinophil-specific marker together with a clear cellular and spatial localization of the Gal-10 signal would be required.

Metachronous CRC showed much lower mRNA expression of Gal-10, when compared with non-metachronous CRC [43]. Patients with overexpression of Gal-10 mRNA more often had CRC without any lymph node or distant metastasis [43]. Moreover, increased prevalence of high expression of Gal-10 mRNA in mucinous adenocarcinomas when compared with conventional adenocarcinomas was reported [43]. However, these results should be interpreted with even more caution, since bulk tissue mRNA analysis cannot distinguish genuine tumor or stromal expression from infiltrating eosinophils.

**Molecular mechanisms of Gal-10 in CRC**

In the context of CLC in CRC, very few studies have been conducted; hence, our knowledge regarding the role of Gal-10 in CRC is very limited. Nevertheless, it was demonstrated that upon macrophage phagocytosis, CLCs activate the NOD-like receptor family pyrin domain containing 3 (NLRP3) inflammasome [217], thereby triggering IL-1β release. Subsequently, NLRP3 activation also triggers the release of IL-18, which in turn activates other eosinophils [217].

Taken together, the involvement of Gal-10 in CRC is largely underexplored. It is likely that tissue Gal-10 levels reflect eosinophilic infiltration, which warrants further studies distinguishing eosinophil-derived from tumor-derived Gal-10. Circulating Gal-10 has been proposed as a potential biomarker for persistent airflow limitation in adult asthmatics [221], but its potential in CRC has not yet been investigated.

### 3.9. Galectin-12

Galectin-12 (Gal-12), a tandem-repeat galectin with two CRDs, was first discovered by mRNA analysis in the Jurkat T cell line [222]. Using an RT-PCR procedure, Gal-12 mRNA was also detected in the heart, pancreas, spleen, thymus, peripheral blood leukocytes, lung, skeletal muscle, kidney, prostate, testis, ovary, colon, and various cancer cell lines [222]. To date, Gal-12 remains one of the most poorly understood galectins, but it was found to suppress cancer cell growth by arresting the cell cycle at the G1 phase (Gal-12-transfected HeLa human cervical cancer cell line), with concurrent downregulation of cyclin A levels and retinoblastoma protein phosphorylation in preliminary studies [222]. Gal-12 was found in nuclei of differentiated, but not undifferentiated, mouse adipocytes and was demonstrated to be involved in PPARγ ligand-induced adipocyte apoptosis [223]. Similar to Gal-10, the role of Gal-12 in CRC is emerging and largely speculative.

**Gal-12 expression patterns in CRC**

According to an observational study, Gal-12 mRNA was downregulated in 72% and overexpressed in 21% of CRCs [43]. Lower expression of Gal-12 mRNA correlated with the smaller size of tumors [43]. Overexpression of Gal-12 was significant in mucinous adenocarcinomas when compared with conventional adenocarcinomas [43]. However, this study was based on a relatively scarce sample size (<100) and should be taken with caution. Another study seems to confirm that Gal-12 mRNA is expressed in vivo in differentiated healthy colon mucosa but significantly decreased in primary tumors [224]. Among CRCs with lymphovascular invasion, Gal-12 showed an increased prevalence of low mRNA expression, but without statistical significance (*p* = 0.051) [43]. CRCs that are characterized by high levels of Gal-12 mRNA tend to have better survival rates. However, the exact *p*-value was not reported [43].

**Mechanistic studies on Gal-12 in CRC**

Studies on nine human colon adenocarcinoma cell lines (LS180, SW480, SW707, HCT15, KM12, TC7, CX1, HT29, LS513) revealed that sodium butyrate, a short-chain fatty acid produced during fermentation by colonic bacteria, induces transcription of the Gal-12 gene in eight of nine cell lines (except for LS513) [144]. It has been proposed that epigenetic alterations may play a role in the loss of Gal-12 expression in CRC. Subsequent studies showed that promoter hypermethylation represses Gal-12 expression in CRC cells, while hypomethylation induces de novo expression of Gal-12 at the transcript level [224]. Studies on molecular signaling pathways involving Gal-12 in CRC revealed a potential Gal-12 protein interactome: SLC3A2, SLC1A5, SLC25A6, VPS13C, TUBB4A, TUBB2A, PHGDH, CDK1, DDX3X, and CD44 [225]. Particularly, Gal-12 was shown to bind the neutral amino acid transporter B(0) (SLC1A5) and significantly suppress glutamine uptake [225]. This suggested that Gal-12 may be a novel inhibitor of glutamine anaplerosis in colon cancer cells, which remarkably diminishes glutamine utilization for energy metabolism and macromolecule synthesis. Loss of Gal-12 expression in CRC tumors appears to mitigate the effects of glutamine deprivation, promoting adaptation for elevated metabolic demands required for tumor growth and progression [225]. It has been speculated that Gal-12 also interferes with the serine synthesis pathway (SSP) activity through glutamine deprivation and binding to the PHGDH enzyme, resulting in decreased glutathione synthesis [225]. Notably, high PHGDH expression in CRC correlates with advanced TNM stage and poor patient outcome [226].

The role of Gal-12 in CRC remains largely unexplored, and the available evidence is limited to a small number of studies. Several promising but unconfirmed hypotheses regarding its molecular pathways have recently emerged. However, the absence of established evidence should not be interpreted as an indication of biological irrelevance, but rather as a need for further mechanistic and clinical studies to elucidate the role of Gal-12 in CRC.

### 3.10. Placental Galectins

Three genes in a chromosome 19 cluster encoding galectin-13 (Gal-13), galectin-14 (Gal-14) and galectin-16 (Gal-16) emerged during primate evolution but remain purely hypothetical in CRC. To date, placental galectins have been researched mainly in the context of pregnancy and pregnancy-associated disorders, owing to their predominant expression in the placenta [227,228]. Gal-13 has emerged as useful in preeclampsia diagnosis, being relatively accurate for identifying true negative cases [229]. Although formerly thought to be expressed solely in the placenta, placental galectins were also demonstrated in other normal tissues, including bladder, spleen, and kidney, as well as in tumor tissues such as liver adenocarcinoma, melanoma and neural tumors [230]. Analyses of public expression databases showed placental galectin expression in breast, lung, testis, ovary, and thyroid cancers [228]. Gal-14 expression was confirmed by Western blot in OVCAR3 ovarian cancer cells and Huh-7 hepatocellular carcinoma cells [231]. Very few studies have investigated the involvement of placental galectins in cancer, despite the fact that pregnancy and prenatal development share several functional features and physiological properties recognized within the hallmarks of cancer [228,231,232,233,234]. To date, no direct study has investigated the role of placental galectins in CRC. However, bioinformatic data from the Gene Expression Omnibus (GEO) database indicate detectable LGALS16 transcript levels in the CRC cell line SW620 (dataset GDS5416) [232]. This observation is hypothesis-generating and has not been independently validated at the RNA or protein level in CRC. Therefore, the potential involvement of placental galectins in CRC remains an open research question requiring experimental validation.

### 3.11. Future Perspectives

Although substantial progress has been made in understanding the role of galectins in CRC, several important questions remain unresolved. One of the major limitations of the current evidence is the considerable heterogeneity among published studies. Differences in patient cohorts, tumor stage and location, molecular characteristics of CRC, sample type (serum, plasma, tissue, or feces), tumor and stromal cellular composition, analytical methodologies, antibody specificity, and experimental models may contribute to inconsistent findings, particularly for Gal-2, Gal-7, Gal-8, and Gal-9. In studies evaluating circulating galectins, differences in sample processing and assay platforms may further affect measured concentrations and limit comparability between studies. Moreover, differences in treatment exposure and inflammatory status may influence galectin levels independently of tumor biology. For example, the discrepancy reported for Gal-4 between tissue expression and circulating concentrations illustrates how differences in biological compartments may affect the interpretation of galectin-based biomarkers. Consequently, direct comparison of studies is often difficult, and the true clinical significance of several galectins remains uncertain.

Current evidence suggests that Gal-1 and Gal-3 are the best-characterized members of the family, emerging as multifunctional regulators of CRC progression. Both proteins are implicated in epithelial–mesenchymal transition, immune evasion, angiogenesis, activation of Wnt/β-catenin and PI3K/AKT signaling, and resistance to anticancer therapy. These implications make them promising candidates for therapeutic targeting, although this remains a hypothesis requiring functional confirmation in physiologically relevant models. Gal-4 also appears particularly interesting because of its predominantly tumor-suppressive properties in CRC, despite elevated circulating concentrations observed in patients. This apparent discrepancy between tissue expression and circulating levels highlights the complexity of galectin biology and warrants further investigation. Similarly, growing evidence indicates that Gal-8 and Gal-9 may influence both tumor progression and antitumor immunity; however, their context-dependent functions remain insufficiently understood.

Despite encouraging results, none of the galectins currently demonstrate sufficient diagnostic or prognostic performance to serve as an independent biomarker in routine clinical practice. Instead, available evidence suggests that their greatest clinical potential may lie in multimarker panels combining galectins with established biomarkers, molecular alterations, or inflammatory mediators. Likewise, if effective galectin-targeting agents emerge from preclinical development, they would likely be most effective when integrated with existing approaches, including chemotherapy, targeted therapy, and immunotherapy.

Future studies should prioritize large, prospective, multicenter clinical cohorts using standardized analytical protocols to validate the diagnostic and prognostic value of circulating and tissue galectins. Mechanistic investigations should further clarify the context-dependent intracellular and extracellular functions of individual galectins, their interactions with the tumor microenvironment, and the molecular determinants responsible for their seemingly contradictory biological effects. Particular attention should also be directed toward elucidating the cooperative and potentially redundant interactions among different galectin family members, as most studies have focused on single proteins despite their overlapping biological activities. Experiments should use siRNA and CRISPR with several independent sequences, together with rescue evaluation. Effects specific to galectins must be distinguished from galectin-independent variables, such as transfection artifacts, cellular stress, or altered proliferation. Appropriate controls should be used, considering recombinant-protein purity and concentration, endotoxin contamination, and glycan-binding activity. When the proposed mechanism may depend on carbohydrate recognition, glycan-binding-deficient mutants would be particularly useful. Moreover, in antibody-based studies, validation of antibody specificity, isotype controls, and secondary-antibody controls should be employed. Findings should be confirmed across various CRC cell lines representing different molecular subtypes, together with normal colorectal epithelial controls. Involvement of galectins in immune regulation should be verified in immune-competent models, organoids with defined stromal and immune components, or primary patient-derived samples. Finally, the development of selective galectin inhibitors and the evaluation of combination therapies targeting galectin-mediated signaling pathways represent promising directions for future translational and clinical research.

Overall, accumulating evidence indicates that galectins are integral regulators of CRC biology, influencing tumor initiation, progression, metastasis, and immune modulation through multiple interconnected signaling pathways. Continued integration of molecular, translational, and clinical research will be essential to determine whether these multifunctional proteins can ultimately be translated into reliable biomarkers and effective therapeutic targets in CRC. However, it is important to emphasize that the current evidence supports the biological relevance of several galectins in CRC pathophysiology but not their readiness for clinical implementation.

## 4. Conclusions

Galectins play an important role in CRC development and progression, especially metastasis, whereas the expression patterns of several galectins vary between normal mucosae, adenomas, and carcinomas. Moreover, these patterns differ heavily, depending on the cell line, changing even within cells of the same patients and the tumor region. This could explain, in part, some discrepancies in galectin expression in the literature.

**Figure 1:**
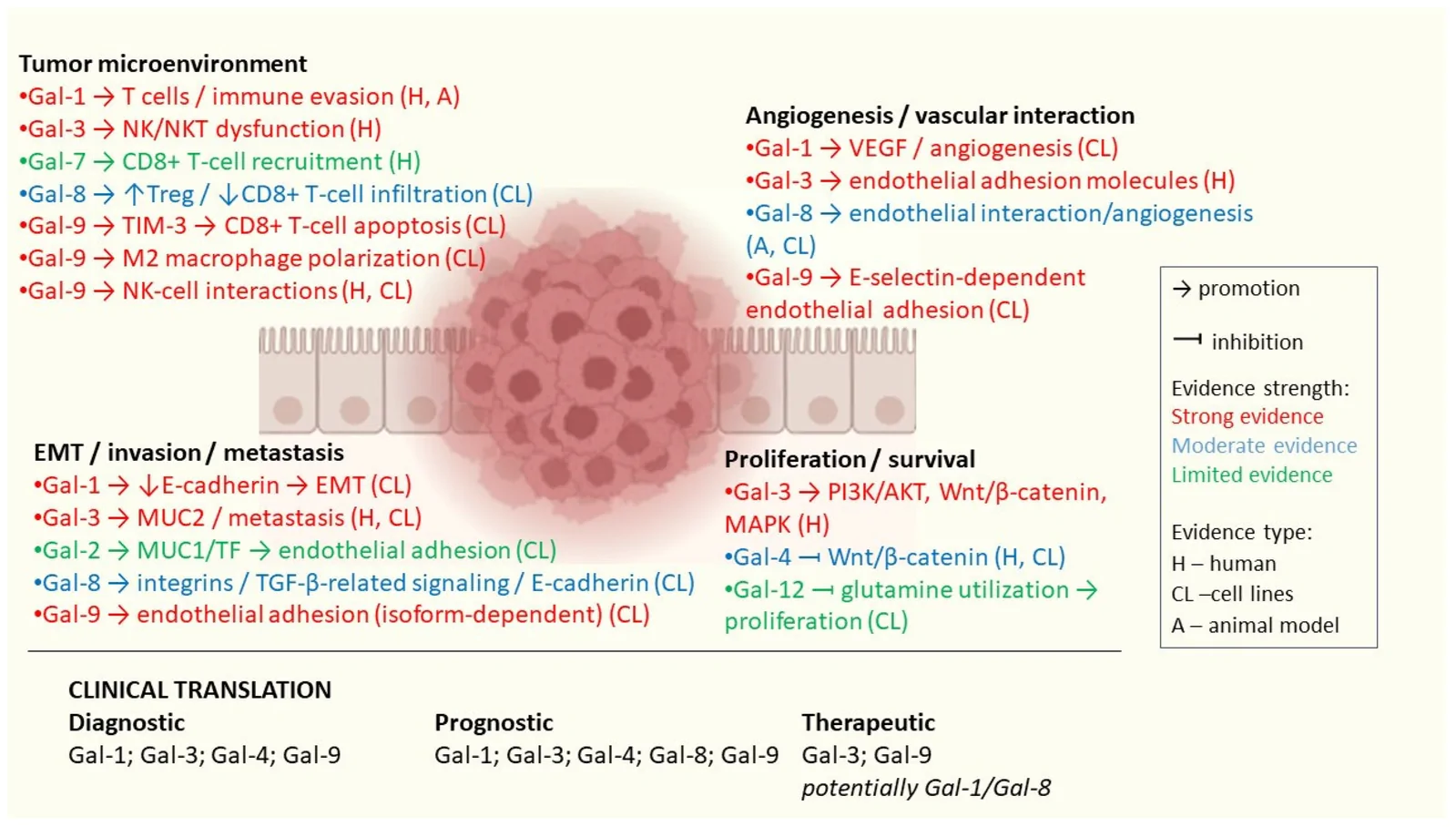
Evidence of the biological and clinical significance of galectins in CRC.

Galectins seem to form a complex network influencing various aspects of cell life by binding to a plethora of ligands and modulating distinct molecular signaling pathways, and the current evidence on the biological and clinical significance of galectins in CRC is summarized in Figure 1. Interestingly, this network shows redundancy at key points in cancer pathophysiology, for example, increased Gal-1 and Gal-3 levels were both shown to enhance the Wnt/β-catenin signaling pathway, presumably by different mechanisms, whereas Gal-4 was shown to attenuate Wnt signaling, and it has been hypothesized that CRC cells shed Gal-4 expression, although the proteolytic mechanism remains undemonstrated. Similarly, multiple galectins facilitate cancer cells’ adhesion capabilities and metastasis formation. Secreted Gal-1 has several direct tumor-promoting effects in CRC, significantly enhancing the invasive capacity and decreasing expression of E-cadherin at the cell junctions. Gal-3 modulates the expression of MUC2 in CRC cells, independently correlating with malignant behavior. Binding of Gal-2 to the TF antigen on the mucin protein MUC1 of tumor cells has been proposed to enhance cancer cell adhesion to the vascular endothelium. Gal-3 also induces the secretion of IL-6, G-CSF, and sICAM-1 from blood vascular endothelial cells, increasing endothelial cell surface adhesion molecules. In Gal-8-overexpressing tumors, higher levels of E-cadherin can be observed. Ectopic Gal-9M and Gal-9S increase E-selectin mRNA expression, enhancing adhesion to endothelial cells, whereas Gal-9L decreases E-selectin mRNA levels. Galectins not only influence cancer cells themselves but also immune cells in the cancer microenvironment, facilitating immune evasion. Gal-1 enhances CD8+ regulatory T cell (Treg) activity, attenuating the antitumor response. Gal-3 expression has been inversely correlated with NKp30 expression on NKT cells. There is evidence that Gal-7 could enhance the cytotoxic immune response by recruitment and activation of CD8+ T cells, although the mechanism remains unknown. Gal-8 upregulates FoxP3-positive Treg cell levels and downregulates CD8+ T cell infiltration. Moreover, secreted Gal-9 was shown to increase the apoptosis of tumor-infiltrating CD8+ T cells. Furthermore, it has been shown that Gal-1 and Gal-3 may play a role in the cell cycle regulation of colon cancer cells. Gal-12 was shown to arrest the cell cycle of HeLa cells, suppressing cancer growth, which would warrant research in the context of CRC cells. Similarly, expression of LGALS16 in the SW620 CRC cell line found in the Gene Expression Omnibus database would warrant experimental validation of Gal-16 potential expression in CRC. An anti-Gal-3 antibody attenuated platelet-induced COX-2 overexpression in vitro, suggesting that Gal-3 blockade may help prevent platelet-induced tumor-promoting signaling. However, this finding requires in vivo validation.

The diagnostic and prognostic value of single circulating galectins is limited. Particularly, the prognostic role of Gal-8 remains inconclusive. However, a multi-biomarker approach may be useful. Simultaneous determination of serum Gal-3 and Gal-4 concentrations resulted in 91% sensitivity and 73% specificity in differentiating patients with and without liver metastases, which is promising, but not clinically validated. Most diagnostic estimates require independent external validation and may be affected by retrospective design, small sample size, or spectrum bias. It is also worth noting that statistical association or discriminative ability does not imply that galectin measurement changes clinical management or improves patient outcomes. Studies that investigate the use of galectins as biomarkers are substantially limited by inconsistent reporting of preanalytical and analytical conditions. Plasma and serum should not be treated as interchangeable. Circulating Gal-3 has been reported to be higher in plasma than in serum [71]. Moreover, few studies systematically account for factors that could alter the results, such as active inflammation, renal function impairment, anemia, and comorbidities that may influence galectin concentrations independently of tumor biology. Technical details, such as sample collection, processing time, storage duration, and freeze–thaw cycles, are also not standardized and rarely fully reported. It is also unclear whether an assay measures total, free, oligomeric, isoform-specific, or biologically active galectin. Taken together, these factors highlight the need for standardization in future studies.

## Figures and Tables

**Table 1:** Evidence on the compartment-dependent role of Gal-1 in CRC.

Reference	Sample	Model	Method	Key Findings
Extracellular/Secreted Gal-1				
[51]	Cell lines Mice	-Syngeneic CT26/CT26-Gal-1-shRNA -AOM-DSS colitis-associated CRC mouse model (LGALS1^−/−^ C57BL/6)	Western blot/Flow cytometry/Suppression assay	Tumor-derived and stromal Gal-1 modulate immunosuppressive activity of CD8+ CD122+ PD-1+ Tregs enhancing tumor growth. (Although mechanistically this appears to be extracellular interaction, it is not directly confirmed in the study)
[53]	Cell lines Mice Human CRC tissues (primary *n* = 40 metastasis *n* = 10 normal colon *n* = 9)	-KM12C/HCT116 -KM12C-WB1 xenograft C57BL/6 and NOD-SCID mice	Western blot/ELISA/ Mass spectrometry/ Exogenous rhGal-1	Stromal-secreted Gal-1 may directly promote EMT, sphere formation, metastasis, and drug resistance through SOX9 and β-catenin.
[54]	Cell lines Mice Human CRC tissues (Stage I *n* = 20 Stage IV *n* = 20) Liver metastasis	-293T/Caco2/HCT116 -HCT116-Gal-1-siRNA -Syngeneic CT26/AS-90K BALB/c mice	IHC/Western blot	Direct interaction between 90K and extracellular Gal-1 attenuates 90K suppression of Wnt/β-catenin.
[56]	Cell lines	-SW620	Western blot/Flow cytofluorimetry	Rac1 knockdown reduces total and surface Gal-1.
[60]	Cell lines	-HCT116/HCT8/HT29 -HCT116-Gal-1-siRNA	Western blot/ELISA/Exogenous rGal-1	LPS-induced TLR4 signaling upregulates Gal-1 secretion in CRC cells, which triggers EMT through activation of ADAM10 and ADAM17.
[62]	Cell lines	-Colo201 -Colo201-Gal-1-transfectants	Immunofluorescence/ Extracellular GST-Gal-1 fusion protein/ Inhibition with lactose	Extracellular Gal-1 may induce CRD-dependent adhesion of CRC cells to ECM.
Intracellular Gal-1				
[61]	Cell lines	-LS180-Gal-1-transfectants -ATRFLOX-Gal-1-siRNA	Immunocytochemistry/ Immunoprecipitation of media/Flow cytometry	Intracellular Gal-1 may induce p21 and apoptosis, with concomitant reduction in cyclin D1, β-catenin/TCF-1/TCF-3 (Wnt) and phospho-IKKα/β/p65 (NF-κB) signaling.
[62]	Cell lines	-Colo201 -Colo201-Gal-1-transfectants	Immunofluorescence/ TUNEL assay/Western blot	Intracellular Gal-1 may be involved in apoptosis and inhibition of CRC cell proliferation.
Compartment not established				
[52]	Cell lines	-KM12C -KM12C-MSC co-culture	cDNA microarray/ RT-qPCR	Direct contact between CRC cells and MSCs upregulates EMT-related gene expression, including Gal-1.
[55]	Cell lines	-SW620-Rac1-transfectants -SW620-Rac1-shRNA	cDNA microarray/ qPCR	Rac1 overexpression activated Wnt signaling and inhibited TGF-β signaling, with concomitant increase in LGALS1 expression in CRC cells.
[57]	Cell lines Human CRC tissues paired with normal colon tissues (*n* = 77)	-HCT8-LYAR-transfectants -HCT116/HCT8-LYAR-siRNA	IHC–LYAR only/Western blot/ qPCR/whole-genome microarray	LYAR promotes CRC migration and invasion through LGALS1 upregulation.
[58]	Cell lines Mice Human CRC tissues (*n* = 470)	-HCT116/SW480 -HCT116/SW480-CHIP-transfectants -HCT116/SW480-CHIP-shRNA -HCT116-xenograft-BALB/c mice	Tissue microarray/IHC/ RT-PCR/Western blot	CHIP-dependent Gal-1 ubiquitination. High CHIP or low Gal-1 expression reduced CRC growth and metastasis in vitro and in vivo.
[59]	Cell lines Human CRC tissues (*n* = 40)	-LS174T/RKO/SW1116/SW620 -SW1116/SW620-HIF-1α-transfectants -SW620-HIF-1α-shRNA -SW620-HIF-1β-shRNA -SW620-Gal-1-shRNA	IHC/RT-qPCR/Western blot	Gal-1 may be a direct target of transcription factor HIF-1. Gal-1 could mediate hypoxia-induced migration and invasion of CRC cells.

## Data Availability

No new data were created or analyzed in this study. Data sharing is not applicable to this article.
